# Efficacy and safety of GLP-1 receptor agonists in patients with metabolic-associated fatty liver diseases: an umbrella review

**DOI:** 10.3389/fmed.2026.1783429

**Published:** 2026-05-28

**Authors:** Hui Wu, Zi-Heng Zhang, Ting Ye, Jia-Ni Chen, Tao-Hsin Tung, Jian-Sheng Zhu

**Affiliations:** 1 Department of Infectious Diseases, Taizhou Hospital, Zhejiang University, Linhai, Zhejiang, China; 2 Department of Infectious Diseases, Taizhou Hospital of Zhejiang Province Affiliated to Wenzhou Medical University, Linhai, Zhejiang, China; 3 Evidence-Based Medicine Center, Taizhou Hospital of Zhejiang Province Affiliated to Wenzhou Medical University, Linhai, Zhejiang, China

**Keywords:** glucagon-like peptide-1 receptor agonists, metabolic disease, metabolic dysfunction-associated steatotic liver disease, public health, umbrella review

## Abstract

Metabolic dysfunction-associated steatotic liver disease (MASLD) is the most common chronic liver disease worldwide Glucagon-like peptide-1 receptor agonists (GLP-1 RAs) play a regulatory roles in metabolism across several tissues and have potential value in the treatment of MASLD. A novel umbrella review was conducted to assess the efficacy and safety of GLP-1 RAs in MASLD in multiple dimensions. Following the PRISMA 2020 guidance, five databases were searched from inception to 30 September, 2025. The quality of the included systematic reviews was assessed using AMSTAR 2. Twenty-three meta-analyses and systematic reviews were included. All outcomes regarding the efficacy of GLP-1 RAs in MASLD were summarized into five categories: liver characteristics, liver enzyme levels, anthropometric measurements, metabolic markers, and inflammatory markers. GLP-1 RAs show potential benefits in improving MASLD across multiple dimensions with a relatively acceptable safety profile, but nearly 80% of included reviews are of critically low quality with high evidence overlap. Thus, our findings are preliminary and hypothesis-generating. High-quality studies are urgently needed.

## 1 Introduction

Metabolic dysfunction-associated steatotic liver disease (MASLD), formerly known as non-alcoholic fatty liver disease (NAFLD), is a growing epidemic associated with metabolic disease (1). Over the last 30 years, owing to the advances in viral liver disease treatment and lifestyle changes, MASLD has become the most common chronic liver disease worldwide, causing a significant burden on healthcare systems (2–4). With the in-depth study of the pathogenesis of MASLD, the concept of multiple-hit mechanisms and the systems view has gradually gained wide acceptance (5). However, its exact pathogenesis remains unclear. Until now, lifestyle interventions, as well as the management of complications such as type 2 diabetes, remain the cornerstone and primary approach for treatment.

Glucagon-like peptide-1 receptor agonists (GLP-1 RAs), including liraglutide, exenatide, semaglutide, beinaglutide, and dulaglutide, are commonly used in clinical practice to manage blood sugar and body weight. They can promote satiety, potentiate insulin secretion, reduce glucagon secretion, and play antiapoptotic and metabolic regulatory roles in several tissues (6, 7). Among then, semaglutide have been conditionally approved by the FDA for the treatment of adults with non-cirrhotic metabolic dysfunction-associated steatohepatitis (MASH) with moderate to advanced fibrosis (8, 9). GLP-1 RAs can modulate hepatic lipid metabolism, inhibit lipogenesis in the liver, reduce steatosis, enhance mitochondrial function, and improve hepatocyte survival (10, 11). GLP-1 RAs show a very promising therapeutic potential in MASLD. Therefore, investigating the efficacy and safety of GLP-1 receptor agonists in patients with MASLD is of great significance.

However, numerous studies assessing the efficacy and safety of GLP-1 RAs in MASLD, controversy remains. For instance, a previous study indicated that GLP-1 RAs significantly decreased the hepatic fat content (HFC) in MASLD, whereas another study found no significant resolution (12, 13). GLP-1 RA treatment had the same effect on liver enzymes in MASLD (14, 15). Although numerous meta-analyses and systematic reviews have demonstrated the resolution of MASLD, considerable heterogeneity exists. Furthermore, the efficacy and safety of GLP-1 RAs in MASLD have not been summarized. MASLD is a systemic metabolic disease associated with multiple indicators, which can be summarized into five key aspects: liver characteristics, liver enzymes, anthropometric measurements, metabolic markers, and inflammatory markers. Additionally, indicators treated with different GLP-1 RAs types exhibit varying efficacies (16, 17). An overarching evidence synthesis is essential to inform clinical guidelines and identify areas that need further research and clinical attention. Umbrella reviews can provide a comprehensive and holistic understanding of an issue by systematically collecting and screening systematic reviews and meta-analyses and providing the highest level of evidence (18). However, few umbrella reviews have explored the efficacy and safety of GLP-1 RAs in MASLD.

Therefore, an umbrella review was conducted to summarize the epidemiological evidence on the efficacy and safety of GLP-1 RAs in MASLD. The efficacies were analyzed and summarized based on these five aspects. The efficacy and safety of different GLP-1 RAs were also explored. This umbrella review aimed to determine (1) whether GLP-1 RAs can relieve the development of MASLD and/or lead to safety problems, and (2) which indicators' resolution and/or safety problems were observed with GLP-1 RAs treatment.

## 2 Methods

An umbrella review is used to synthesize and evaluate evidence from existing systematic reviews and meta-analyses, providing a comprehensive and holistic understanding of an issue and the highest level of evidence (19, 20). This review complied with the Preferred Reporting Items for Systematic Reviews and Meta-Analyses 2020 statement and was registered with the International Prospective Register of Systematic Reviews (ID: CRD420251089535) (21).

### 2.1 Search strategy

References for this review were identified by searching PubMed, Embase, the Cochrane Library, Web of Science, and Scopus for related studies from the inception to September 30, 2025. The substantially higher yield from Scopus reflects its broader coverage of journals and document types; however, after screening only eligible systematic reviews were included, minimizing any impact on the final synthesis. The search strategy was developed according to the populations-interventions-comparators-outcome (PICO) criteria and included key and MESH terms related to MASLD patients, GLP-1 RAs, the placebo or standard of care of MASLD and the efficacy and safety of GLP-1RAs. The keywords, “Glucagon-Like Peptide-1 Receptor Agonists” and “Metabolic Dysfunction-Associated Steatotic Liver Disease” were used to identify the relevant studies. No other restrictions were imposed. Two researchers independently performed the searches, and any disagreements were resolved through discussion with a third author. Details of the search strategy are presented in Supplementary Table S1.

### 2.2 Study screening

Meta-analyses and systematic reviews that reported the effects of GLP-1RAs on MASLD were included. The inclusion criteria were as follows: (1) meta-analyses and systematic reviews; (2) the population was diagnosed with MASLD based on liver histology biopsy or imaging examination; (3) the intervention measures involved GLP-1RAs; and (4) the outcomes included the efficacy (liver enzyme levels, metabolic markers, hepatic fat content, and others) and/or safety [occurrence of nausea, constipation, diarrhea, and other adverse events (AEs)] of GLP-1RAs compared to the placebo or standard of care. The exclusion criteria were as follows: (1) network meta-analyses; (2) not formally published papers, including conference abstracts, commentaries, and editorials; (3) the full text or complete data of studies were unavailable; (4) patients not diagnosed with MASLD; and (5) not treated with GLP-1RAs.

The articles were imported into EndNote 20. Duplicates were excluded. Additionally, based on titles and abstracts, the retrieved studies were manually screened according to the inclusion and exclusion criteria. The full text was reviewed to ensure conformance with the subject and comprehensive data acquisition. Two researchers independently screened the studies, and any disagreements were resolved through discussion with a third author.

### 2.3 Data extraction

Data extraction from the eligible studies was independently performed by two authors, including year; author; evidence reviewed; number of primary studies; population; sample size; study design; GLP-1 RA type and dose; control group; follow-up time; age; rate and number of males; body mass index (BMI); outcome for efficacy and AEs; and meta-analysis (MA) models.

### 2.4 Data synthesis

The available data from the included studies and their supplementary files were presented comprehensively to synthesize the existing evidence from the selected eligible systematic reviews and meta-analyses. Pooled estimates derived from the incorporated meta-analyses are illustrated in a forest plot.

Multiple primary studies were included. To account for the extent of multiple inclusion of primary studies (overlaps) in the included systematic reviews, the Corrected Covered Area (CCA) formula was used:

$$\text{CCA=}\frac{\text{N-r}}{\text{rc-r}}$$

where N is the sum of the included primary studies, accounting for double counting, r is the number of distinctly indexed primary studies, and c is the number of included systematic reviews (22). A CCA score ≤ 5% implies a slight overlap, 6%−10% indicates a moderate overlap, and 11%−15% or >15% suggests a high or very high overlap. The overlap of primary studies is inevitable in multiple systematic reviews. A higher degree of overlap suggests that the synthesized evidence in umbrella reviews is predominantly derived from multiple review studies that rely extensively on the same primary studies (23).

### 2.5 Quality assessment

AMSTAR-2 guidelines were used to assess the methodological quality of the included systematic reviews and meta-analyses. Included studies were graded as high, moderate, low, or critically low. The guidelines contained 16 terms, with seven items considered critical. Any critical domain with deficiencies can affect the overall effectiveness of the review (24, 25).

### 2.6 Credibility assessment of evidence and methods

In clinical practice, recommendations are stronger when supported by stronger evidence. High epidemiological reliability denotes the strongest available evidence with no indication of significant variance or bias (26). The following categories were used to classify the level of evidence in the included studies (27):

(i) Persuasive: statistical significance per the random-effects model of *p* < 0.000001, >1,000 cases, low heterogeneity among the selected studies (*I*^2^ < 50%), 95% confidence interval (CI; excluding the null value), and no evidence of small study effects or significant bias.
(ii) Highly recommended: statistical significance of *p* < 0.000001, >1,000 cases, and most studies indicating a significant effect.
(iii) Recommended: >1,000 cases with significant effects at *p* < 0.001.
(iv) Weak evidence: nominally significant association (*p* < 0.05).
(v) Poor evidence: obtained from samples with < 1,000 cases.

## 3 Results

### 3.1 Study search and selection

**Figure 1:**
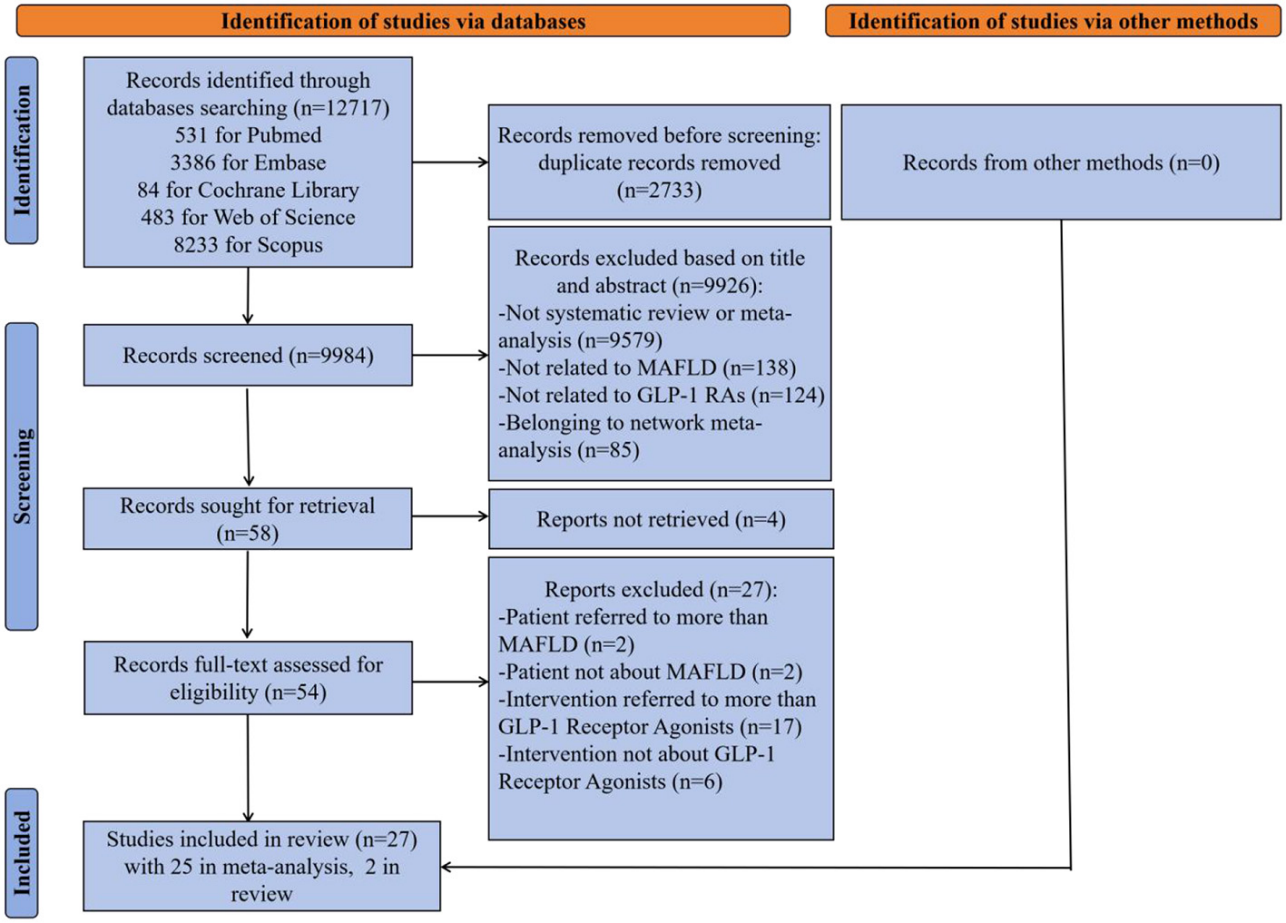
Flow diagram for the selection of included studies.

A total of 12,717 records were retrieved, including 531 from PubMed, 3,386 from Embase, 84 from the Cochrane Library, 483 from Web of Science, and 8,233 from Scopus, the details of the search strategy was shown in Supplementary Table S1. After excluding duplicated articles and applying the eligibility criteria, finally, 27 articles including 25 meta-analyses and two systematic reviews with 62 distinct pooled analyses were finally included (Figure 1 and Supplementary Table S2).

### 3.2 Characteristics of included reviews

Characteristics of the systematic reviews and meta-analyses are presented in Table 1. The 27 studies were published between 2016 and 2025 (Supplementary Figure S1). These reviews involved five types of GLP-1 RAs; liraglutide was discussed in 24 reviews, exenatide in 15 reviews, semaglutide in 12 reviews, beinaglutide in one review, and dulaglutide in seven reviews (Supplementary Figure S2). Most reviews summarized the efficacy outcomes of GLP-1 RAs from five aspects, (a) liver characteristics, such as histological resolution (defined as the disappearance of hepatocyte ballooning without worsening fibrosis), degree of liver fibrosis [Fibrosis-4 (FIB-4), enhanced liver fibrosis test score, and NAFLD fibrosis score], NAFLD activity score and HFC; (b) the serum liver enzymes: alanine aminotransferase (ALT), aspartate aminotransferase (AST), gamma-glutamyl transferase (GGT) and alkaline phosphatase (ALP); (c) anthropometric measurements, such as body weight, BMI, waist circumference (WC), waist-to-hip ratio, visceral adipose tissue (VAT), and subcutaneous adipose tissue (SAT); (d) metabolic markers, including high density lipoprotein, low density lipoprotein (LDL), glycated hemoglobin A1c (HbA1c), and fasting blood glucose (FBG); and (e) inflammatory markers like c-reactive protein (CRP), interleukin-6, and cytokeratin-18. Most reviews also assessed GLP-1 RAs. The number of primary studies included in each meta-analysis and systematic review varied from three to 27. Most of the primary studies included in the 23 reviews were randomized controlled trials, although some also included observational cohort and retrospective studies. The 23 reviews included 51 unique primary studies, with a high overlap of 10.47% (Supplementary Table S3). Primary studies from several countries and regions, including the UK, USA, Singapore, China, Japan, Netherlands, Finland, Canada, Denmark, France, India, Greece, Italy, and Spain, were used (shown in Supplementary Figure S3 and Supplementary Table S4).

**Table 1:** Characteristics of included studies.

No.	References	Evidence reviewed	Number of studies	Population (*N*)	Study designincluded	Kind of GLP-1 RAsand dose	Control group	Follow-up (weeks)	Male, % (*n*)	Mean age (years)	BMI (kg/m^2^)	Efficacy outcomes	Adverse effects	Meta-analysesmodels	Study quality
1	Potter et al. (51)	Inception to November 16, 2023	6 (3 about liraglutide; 3 about semaglutide)	NAFLD/ MASLD (443)	RCT	Liraglutide: 1.8 mg qd/3 mg qd; Semaglutide: 0.4 mg qd/2.4 mg qw.	The standard care or placebo	26–42	31(22)− 90 (27)	40.7–59.5	NA	**The primary outcome:** (a) **The resolution of steatohepatitis without worsening of fibrosis:** OR 4.45, 95% CI [1.92, 10.3], *p* = 0.21, *I^2^* = 35% (in favor of GLP-1RAs). **The secondary outcomes:** (a) **Anthropometric measurements:** Body weight [MD = −4.77 kg, 95% CI (−1.51,−2.51), *p* < 0.00001, *I^2^* = 89%]; BMI (MD = −1.2 kg/m^2^, 95% CI (−2.41, 0.01), *p* ≤ 0.00001, *I^2^*= 85%); All in favor of GLP-1RAs. (b) **Liver enzyme:** ALT [MD = −11.81 U/L, 95% CI [−22.18, −1.45], *p* < 0.0001, *I^2^* = 83%]; AST [MD = −7.8 U/L, 95% CI (−15.49, −0.12), *p* = 0.0006, *I^2^* = 79%]. (c) **Characteristics of liver:** The change of GLP-1RAs group in steatosis by MRI-PDFF [MD = −5.09% , 95% CI (−7.49, −2.69), *p* = 0.0004, *I^2^* = 84%]; The change of GLP-1RAs group in liver stiffness by MRE [MD = −0.17 kPa, 95% CI (−0.34, 0), *p* = 0.21, *I^2^* = 36%]; All in favor of GLP-1RAs; Non-significant change in fibrosis stage on biopsy.	Serious adverse events [OR 1.45, 95% CI (0.73, 2.89)]	Random-effects model	Low
2	Huang et al. (34)	Inception to March 2024	10 (1 about beinaglutide, 5 about liraglutide, 4 about semaglutide)	MASLD (960)	RCT	Beinaglutide: 0.1 mg; Liraglutide: 1.2 mg, 1.8 mg, 3 mg; Semaglutide: 0.1 mg, 0.2 mg, 0.4 mg, 1 mg, 2.4 mg.	The placebo or active agents group	24–72	NA	38.6 ± 8.2–60.5 ± 8.5	27.1 ± 3.8–37.7 ± 6.2	NA	Gastrointestinal adverse events; Short-term treatment with GLP-1RA may lead to a higher occurrence of adverse events (< 30 week vs. >30 week)	Random and fixed- effects model	Critically low
3	Xu et al. (13)	Inception to December 1, 2023	12 (All about liraglutide)	NAFLD combined with T2DM (596)	RCT	Liraglutide: 0.6–1.2 mg qd/ 0.9 mg qd/1.2 mg qd/1.8 mg qd.	The placebo or active agents group	12–36	NA	43.1–65.8	Liraglutide: 27.6–40.7 Control: 26.82–41.6	(a) **Characteristics of liver:** Liraglutide induced a non-significant impact on HFC [MD = −1.18, 95% CI (−2.85, 0.49), *p* = 0.17]. (b) **Anthropometric measurements:** Liraglutide induced a significantly reduction on the BMI [MD = −1.06, 95% CI (−1.41, −0.70), *p* < 0.001], VAT [MD = −21.06, 95% CI (−34.58, −7.55), *p* = 0.002], and SAT [MD = −20.53, 95% CI (−29.15, −11.90), *p* < 0.001]. (c) **Liver enzyme:** Liraglutide induced a non-significant impact on AST [MD = −1.09, 95% CI (−2.79, 0.62), *p* = 0.21], but a non-significant impact on ALT [MD = −3.06, 95% CI (−6.17, 0.05), *p* = 0.05], GGT [MD = −0.93, 95% CI (−4.24, 2.38), *p* = 0.58], and ALP [MD = −0.93, 95% CI (−2.93, 1.07), *p* = 0.36]. (d) **Metabolic markers:** Liraglutide induced a significantly reduction on HbA1c [MD = −0.40, 95% CI (−0.65, −0.15), *p* = 0.002] but a non-significant impact on TC levels [MD = −0.10, 95% CI (−0.27, 0.07), *p* = 0.24], TG [MD = −0.35, 95% CI (−0.61, −0.09), *p* = 0.0009], HDL [MD = −0.01, 95% CI (−0.05, 0.03), *p* = 0.59], and LDL [MD = 0.03, 95% CI (−0.17, 0.23), *p* = 0.80].	Gastrointestinal disorder	Random-effects model	Critically low
4	Njei et al. (16)	Inception to January 10, 2024	27 (1 about dulaglutide; 5 about exenatide; 14 about liraglutide; 5 about semaglutie; 1 about tirzepatide; 1 about dulaglutide, exenatide and liraglutide)	MASLD (1,003)	RCT	Dulaglutide: 0.75–1.5 mg qw; Exenatide: 5 ug−10 mg bid; Liraglutide: 0.6–3 mg qd; Semaglutie: 0.05–0.4 mg qd/2.4 mg qw; Tirzepatide: 5–15 mg qd.	The placebo or active agents group	12–72	NA	NA	NA	(a) **Anthropometric measurements:** A significant decrease on body weight [MD = −1.04, 95% CI (−1.28, −0.81), *p* < 0.0001, in favor of GLP-1RAs], liraglutide exhibited a positive mean difference in body weigh change compared to the overall effect, with MD = 0.85, 95% CI [(0.50, 1.20]; A significant improvement on BMI [MD = −0.74, 95% CI (−0.86, −0.63), *p* < 0.0001, in favor of GLP-1RAs]; A significant decrease on WC [MD = −5.64, 95% CI (−6.32, −4.97), *p* < 0.00001, in favor of GLP-1RAs]; A significant reduction on subcutaneous fat [MD = −5.43, 95% CI (−5.61, −5.25), *p* < 0.0001, in favor of GLP-1RAs]. (b) **Liver enzyme:** Significant reduction on ALT [MD = −4.62, 95% CI (−6.64, −2.60), *p* < 0.0001], AST [MD = −0.21, 95% CI (−0.38, −0.04), *p* < 0.0001], and GGT [MD = −0.71, 95% CI (−0.94, −0.47), *p* < 0.0001]. (c) **Metabolic markers:** Significant improvement on diastolic blood pressure [MD = −1.80, 95% CI (−8.30, 4.70), *p* = 0.59], FBG [MD = −0.2, 95% CI (−0.38, −0.02), *p* = 0.03], HbA1c [MD = −0.33, 95% CI (−0.41, −0.25), *p* < 0.0001], HDL [MD = −0.07, 95% CI (−0.09, −0.05), *p* < 0.0001], LDL [MD = −0.12, 95% CI (−0.18, −0.05), *p* = 0.0002], TC [MD = −3.89, 95% CI (−9.71, 1.92), *p* = 0.19], and TG [MD = −0.12, 95% CI (−0.20, −0.05), *p* = 0.001]. (d) **Characteristics of liver:** A significant reduction on HFC [MD = −1.38, 95% CI (−1.50, −1.26), *p* < 0.0001], and non-significant improvement on the FIB-4 score [MD = −0.04, 95% CI (−0.15, 0.08), *p* = 0.5].	Gastrointestinal adverse events, dizziness, flatulence, eructation, headache, fatigue, early satiety, temporary rash at the injection site, hypoglycemia, headache, toothache, daytime urinary frequency.	Fixed-effects model	Critically low
5	Fang et al. (52)	NA	16 (1 about dulaglutide; 3 about exenatide; 10 about liraglutide; 2 about semaglutie)	NAFLD/ NASH (2,178)	RCT	Dulaglutide: 1.5 ug qw; Exenatide: 10 ug qd; Liraglutide: 0.6 mg qd/1.2 mg qd/1.8 mg qd//3 mg qd; Semaglutie: 0.1 mg qd/0.2 mg qd/0.4 mg qd.	The placebo or active agents group	12–72	57.79 (1,258)	38.6 ± 8.2–60 ± 6	26.8 ± 0.7–36.6 ± 6.4	**The primary outcome:** (a) **The resolution of steatohepatitis without worsening of fibrosis:** No significant differences in GLP-1RAs group **[**pooled random-effects OR 1.50, 95% CI (0.98, 2.28), Z-test = 1.88, *p* = 0.06]; A significantly greater histologic resolution in liraglutide and semaglutide groups [pooled random-effects OR 4.08, 95% CI (6.56, 22.54), Z-test = 5.82, *p* < 0.0001, *I^2^* = 0%]. **The secondary outcomes:** (a) **Anthropometric measurements:** Body weight [WMD = −1.93, 95% CI [−3.01, −0.85], *p* = 0.0005], in favor of GLP-1RAs. (b) **Liver enzyme:** A significant decrease in ALT level [WMD = −11.82, 95% CI (−11.70, −6.94), *p* < 0.00001], AST level [WMD = −5.22, 95% CI (−9.07, −1.37), *p* = 0.008], and GGT [WMD = −13.68, 95% CI (−16.88, −10.47), *p* < 0.00001]. (c) **Inflammatory markers:** A significant decrease in CRP level [WMD = −0.41, 95% CI (−0.78, −0.04), *p* = 0.002].	NA	Random- effects model	Critically low
6	Malik et al. (14)	Inception to October 2020	5 (All about liraglutide)	NASH (180)	RCT	Liraglutide	The placebo	NA	NA	Liraglu-tide: 56.2 ± 9.18; Control: 57.7 ± 9.26.	Liraglutide: 32.97 ± 3.8; Control: 33.4 ± 4.	(a) **Anthropometric measurements:** No significant difference on BMI [MD = −0.26, 95% CI (−1.68, 1.16), *p* = 0.72, *I^2^* = 22%]; A significant difference on WC [MD = −10.96, 95% CI (−16.79, −5.14), *p* = 0.02, *I^2^* = 0%]. (b) **Liver enzyme:** No significant difference on ALT [MD = 2.66, 95% CI (−1.56, 6.87), *p* = 0.22, *I^2^* = 0%]; No significant difference on AST [MD = −1.99, 95% CI (5.70, 1.72), *p* = 0.29, *I^2^* = 43%]; A significant difference on GGT [MD = 5.02, 95% CI (−0.86, 10.90), *p* = 0.09, *I^2^* = 45%]; No significant difference on ALP [MD = −5.16, 95% CI (−11.90, 1.59), *p* = 0.13, *I^2^*= 22%]. (c) **Metabolic markers:** No significant difference on TC [MD = −0.31, 95% CI (−0.65, 0.03), *p* = 0.07, *I^2^* = 0%]; No significant difference on TG [MD = −0.14, 95% CI (−0.53, 0.25), *p* = 0.48, *I^2^* = 0%]; A significant difference on HDL [MD = 0.10, 95% CI (0.02, 0.18), *p* = 0.02, *I^2^* = 8%]; A significant difference on LDL [MD = −0.29, 95% CI (−0.56, −0.02), *p* = 0.04, *I^2^* = 0%]; A significant difference on HbA_1c_ [MD = −0.62, 95% CI (−0.88, −0.36), *p* < 0.01, *I^2^* = 30%].	NA	Random-effects model	Critically low
7	Zhao et al. (30)	Inception to March 2022	16 (all about liraglutide)	NAFLD combined with T2DM (1,294)	RCT	Liraglutide: 0.9 mg, 1.2 mg, 1.8 mg qd.	The standard care or placebo	8–36	58.66% (759)	NA	NA	(a) **Liver enzyme:** ALT [MD = −0.99, 95% CI (−1.51, −0.46), *p* = 0.0002]; AST [MD = −0.52, 95% CI (−1.11, 0.08), *p* = 0.09). (b) **Metabolic markers:** HbA1c [MD = −0.9, 95% CI (−1.38, −0.42), *p* = 0.0002], FBG [MD = −1.35, 95% CI (−1.97, −0.77), *p* < 0.00001], TG [MD = −0.66, 95% CI (−0.96, −0.37), *p* < 0.0001], TC [MD = −0.1, 95% CI (−1.56, −0.43), *p* = 0.0006], and LDL-C [MD = −0.28, 95% CI (−0.52, −0.03), p = 0.03].	AE [OR 2.53, 95% CI (1.57, 4.07), *p* = 0.0001]	Random-and fixed-effects model	Critically low
8	Zhu et al. (33)	Inception to May 1, 2023	3 (All about semaglutide)	NASH (458)	RCT	Semaglutie: 0.1 mg qd/0.2 mg qd/0.4 mg qd/2.4 mg qw.	The placebo	48–72	42.36 (194)	55–60	NA	(a) **Characteristics of liver:** Semaglutide has shown a significantly higher likelihood of NASH resolution with no worsening of liver fibrosis [OR 3.18, 95% CI (1.70, 5.95), *I^2^*= 0%]; No significant improvement in liver fibrosis stage without worsening of NASH [OR 0.71, 95% CI (0.15, 3.41, *I^2^*= 80%); Significant improvements in NAS] components with [OR 2.83, 95% CI (1.19, 6.71), *I^2^*= 57%] for steatosis, [1.81, 95% CI (1.11, 2.96), *I^2^*= 0%] for lobular inflammation, and [2.92, 95% CI (1.83, 4.65), *I^2^*= 0%] for hepatocellular ballooning; A significant reduction in liver stiffness on MRE or fibroscan [MD = −0.48, 95% CI (−0.86, −0.11), *I^2^* = 57%]; A significant reduction in liver steatosis on MRI-PDFF [MD = −4.96, 95% CI (−9.92, 0.01), *I^2^* = 64%]. (b) **Anthropometric measurements:** A significant reduction on Body weight [MD = −6.53, 95% CI (−11.21, −1.85), *I^2^*= 0%]. (c) **Liver enzyme:** A significant reduction of ALT [MD = −14.06, 95% CI (−22.06, −6.07), *I^2^* = 0%] and AST = −11.44, 95% CI (−17.23, −5.65), *I^2^* = 0%]. (d) **Metabolic markers:** A significant reduction on HgA1c [MD = −0.77%, 95% CI (−1.18, −0.37), *I^2^* = 75%], TG [MD = −24.03, 95% CI (−60.94, 12.88), *I^2^* = 42%], TC [MD = −7.31, 95% CI (−51.66, 37.03), *I^2^* = 87%], HDL [MD = −7.52, 95% CI (−49.32, 34.27), *I^2^* = 84%], and LDL [MD = −4.72, 95% CI (−56.23, 46.79), *I^2^* = 92%].	Semaglutide has shown a higher occurrence of gastrointestinal related side effects compared to placebo [OR 3.72, 5% CI (1.68, 8.23), *I^2^* = 49%].	Random-effects model	Critically low
9	Dutta et al. (28)	Inception to March 2022	4 (All about semaglutide)	MASLD and/or T2DM (2,115)	RCT	Semaglutide: 0.4 mg qw/0.5 mg qw.	The placebo	59–106	NA	48 ± 13–64.6 ± 7.3	32.7 ± 6.29–39.9 ± 8.8	(a) **Characteristics of liver:** Semaglutide has significantly lower liver stiffness [MD = −3.19 kPa, 95% CI (−3.26, −3.12), *p* < 0.01, fibroscan ^®^] and steatosis [MD = −13.40 dB/m, 95% CI (−20.56, −6.24), *p* < 0.01, fibroscan ^®^]; Non-significant effect on ELFTS [MD = −0.35, 95% CI (−0.80, −0.10), *p* = 0.13, *I^2^* = 78%]. (b) **Anthropometric measurements:** Semaglutide had a significantly reduction on body weight percent reduction [MD = −8.99%, 95% CI (−14.64, −3.34), *p* = 0.002, I^2^ = 100%]. (c) **Liver enzyme:** Semaglutide had a significantly reduction on ALT [MD = −3.89, 95% CI (−5.41, −2.36), *p* < 0.01, *I^2^* = 0%]. (d) **Metabolic markers:** Semaglutide had significantly reduction on HbA1c [MD = −0.77%, 95% CI (−1.10, −0.45), *p* = 0.002, *I^2^* = 100%], TG [MD = −21.43, 95% CI (−41.63, −1.23), *p* = 0.04, *I^2^* = 99%], TC [MD = −5.53, 95% CI (−8.45, −2.61), *p* < 0.01, *I^2^* = 0%], and LDL [MD = −3.55, 95% CI (−5.87, −1.23), *p* < 0.01, *I^2^* = 0%].	Gastrointestinal disorder	Random-effects model	Critically low
10	Bandyo-padhyay et al. (29)	Inception to June 30, 2023	8 (All about semaglutide)	NAFLD/ NASH (2,413)	RCT, single-arm quasi-experi-mental studies	Semaglutide: 0.1, 0.2, 0.4 mg qd/0.5 mg qw/1 mg qw/2.4 mg qw/14 mg .	The placebo or active agents group	24–72	29 (21)−70.1 (72)	29 (21)−70.1 (72)	29.6 (27.4–33.7)−38.8 ± 8.3	(a) **Characteristics of liver:** Semaglutide significantly improved HFC [MD = −4.97%, 95% CI (−6.65,−3.29), *p* < 0.001, *I^2^* = 90%] and liver stiffness [MD = −0.96 , 95% CI (−1.87, −0.04), *p* = 0.04, *I^2^* = 98%]. (b) **Liver enzyme:** Semaglutide significantly improved ALT [MD = 14.07, 95% CI (−19.39, −8.75), *p* < 0.001, *I^2^*= 98%], AST [MD = −6.89, 95% CI (−9.14, −4.63), *p* < 0.001, *I^2^*= 91%], and GGT [MD = −16.17, 95% CI (−28.12, −4.22), *p* = 0.008, *I^2^*= 95%]. (c) **Metabolic markers:** Semaglutide significantly improved HbA1C [MD = −1.02%, 95% CI (−1.24, −0.80), *p* < 0.001, *I^2^*= 94%], TC [MD = −10.70, 95% CI (−18.00, −3.40), *p* = 0.004, *I^2^* = 71%], VLDL-C [MD = −6.13, 95% CI (−9.00, −3.26), *p* < 0.001, *I^2^* = 83%], and TG [MD = −27.03, 95% CI (−35.30, −18.76), *p* < 0.001, *I^2^* = 54%]; But non-significant reduction in the LDL-C [MD = −5.94 , 95% CI (−13.14, −1.51), *p* = 0.12, *I^2^* = 81%], and HDL-C [MD = −0.14 mg/d, 95% CI (−1.44, −1.15), *p* = 0.83, *I^2^*= 35%].	Gastrointestinal disorder	Random-effects model	Critically low
11	Borodav-kin et al. (53)	From 1 January, 2000 to 20 March, 2021	4 (1 about dulaglutide; 2 about liraglutide; 1 about semaglutie)	NASH combined with T2DM (406)	RCT and observa-tion cohort study	Dulaglutide: 0.75mg qd; Liraglutide: 0.9 mg qd/1.8 mg qd; Semaglutie: 0.1 mg qd/0.2 mg qd/0.4 mg qd.	The placebo or lifestyle interven-tion	12–64	41.3 (171)	50 ± 11–66.8 ± 2.7	NA	(a) **Liver Histology:** A decrease in the overall NAS Score [RR = 2.67, 95% CI (1.87, 3.81)]; Improvement of hepatic fibrosis by at least 1 point according to the Kleiner classification [RR = 1.29, 95% CI (0.99, 1.70)]; Improvement of lobular inflammation [RR = 1.44, 95% CI (1.11, 1.86)]. (b) **Liver enzyme:** ALT level (SMD = −0.22, 95% CI (−0.36; −0.09), *p* = 0.87, *I^2^* = 0%]; AST level [SMD = −0.13, 95% CI (−0.20; −0.06), *p* = 0.99, *I^2^* = 0%]; GGT level [SMD = −0.33, 95% CI (−0.56; 0.10), *p* = 0.54, *I^2^* = 0%]. (c) **Other secondary NASH markers:** A significant effects of GLP-1RAs on CCCK-18 M30 [SMD = −1.22, 95% CI (−2.34; −0.10), *p* < 0.01, *I^2^* = 86%].	Gastrointestinal complications	Random-effects model	Low
12	Song et al. (54)	NA	11 (All about liraglutide)	NAFLD combined with T2DM (535)	RCT	Liraglutide: 0.6–1.2 mg qd/0.9 mg qd/1.2 mg qd/1.8 mg qd.	The placebo or active agents group	12–96	NA	NA	NA	(a) **Anthropometric measurements:** A significant decrease on BMI [MD = −1.13 kg/m^2^, 95% CI (−2.03, −0.23), *p* = 0.01, *I^2^* = 83%]. (b) **Liver enzyme:** No significant difference on ALT, AST, GGT, and ALP. (c) **Metabolic markers:** No significant difference on HDL and LDL; Significant decrease on HbA1c [MD = −0.86, 95% CI (−1.22, −0.51), *p* < 0.001; *I^2^* = 67%]; TC [MD = −0.34 mmol/L, 95% CI (−0.65, 0.03], *p* = 0.03, *I^2^* = 28%), and TG [MD = −0.29 mmol/L, 95% CI (−0.57, −0.01), *p* = 0.04; *I^2^* = 25%]. (d) **Characteristics of liver:** No significant difference on LF, SAT, and VAT.	Gastrointestinal complications	Random-effects model	Critically low
13	Zhu et al. (55)	Inception to July 10, 2021	8 (1 about dulaglutide; 2 about exenatide; 5 about liraglutide)	NAFLD combined with T2DM (563)	RCT	Dulaglutide: max 1.5 mg qw; Exenatide: max 10 ug bid/19.43 ± 2.36 ug qd; Liraglutide: max 1.8 mg qd.	The placebo	12–26	26.46 (149)	NA	25.1 ± 1.1–32.8 ± 1.0	(a) **Characteristics of liver:** Non-significant improvement on the FIB-4 index [WMD = −0.04, 95% CI (−0.15, 0.08), *p* = 0.50, *I^2^* = 44%] and the NFS value [WMD = −0.16, 95% CI (−0.56, 0.24), *p* = 0.42, *I^2^* = 0%). (b) **Anthropometric measurements:** Significant improvement on body weight [WMD = −3.48, 95% CI (−4.58, −2.37), *p* < 0.00001, *I^2^*= 55%], BMI [WMD = −1.07, 95% CI (−1.35, −0.78), *p* < 0.00001, *I^2^*= 35%), and WC [WMD = −3.87, 95% CI (−5.88, −1.86), *p* = 0.0002, *I^2^*= 77%]; Significant improvement on SAT [WMD = −28.53, 95% CI (−68.09, −26.31), *p* < 0.00001, *I^2^* = 29%] and VAT [WMD = −29.05, 95% CI (−42.90, −15.9), *p* < 0.0001, *I^2^* = 80%]. (c) **Liver enzyme:** Significant improvement on ALT [WMD = −3.82, 95% CI (−7.04, −0.60), *p* = 0.02, *I^2^* = 58%], AST [WMD = −2.4, 95% CI (−4.55, −0.25), *p* = 0.03, *I^2^* = 49%], and GGT [WMD = −3.38, 95% CI (−8.73, 1.96), *p* = 0.21, *I^2^* = 56%]. (d) **Metabolic markers:** Significant improvement on FBG [WMD = −0.35, 95% CI (−0.06, −0.05), *p* = 0.02, *I^2^* = 13%], HbA1c [WMD = −0.39, 95% CI (−0.56, −0.22), *p* < 0.00001, *I^2^* = 5%], HoMA-IR [WMD = −1.51, 95% CI (– 0.87, −0.16), *p* = 0.005, *I^2^*= 92%, TC [WMD = −0.31, 95% CI (−0.48, −0.13), *p* = 0.0008, *I^2^*= 22%], TG [WMD = −0.27, 95% CI (−0.43, −0.11), *p* = 0.0008, *I^2^*= 0%], and SBP [WMD = −2.52, 95% C I (−5.40, 0.36), *p* = 0.09]; Non-significant improvement on PBG [WMD = −0.67, 95% CI (−1.99, 0.64), *p* = 0.32, *I^2^* = 64%], LDL [WMD = −0.07, 95% CI (−0.26, 0.13), *p* = 0.49, *I^2^* = 51%], HDL [WMD = −0.03, 95% CI (−0.08, 0.02), *p* = 0.28, I*^2^* = 30%], and DBP [WMD = 1.51, 95% CI (−0.77, 3.80), *p* = 0.19, *I^2^* = 0%].	Gastrointestinal discomfort included mainly nausea, vomiting and diarrhea; headache.	Random-effects model	Low
14	Rezaei et al. (15)	Inception to January 2020	10 (3 about exenatide; 7 for liraglutide)	NAFLD/ NASH (677)	RCT	Exenatide; Liraglutide.	The placebo or active agents group	12–48	NA	38.6 ± 8.2–56.4 ± 8.4	NA	(a) **Liver enzyme:** Treatment with GLP-1 RAs lead to the amelioration of ALT [WMD = −10.14, 95% CI (−15.84, −4.44), *p* < 0.001], GGT [WMD = −11.53, 95% CI (−15.21, −7.85), *p* < 0.001], and ALP [WMD = −8.29, 95% CI (−11.34, −5.24), *p* < 0.001]. Non-significant improvement on AST [WMD = −2.95, 95% CI (−7.26, 1.37), *p* < 0.18]. (b) **Metabolic markers:** GLP-1 RAs did not cause a statistically significant change in TG [WMD = −7.07, 95% CI (−17.51, 3.37), *p* = 0.18], TC [WMD = −1.17, 95% CI (−5.25, 2.91), *p* = 0.57], LDL-C [WMD = −1.67, 95% CI (−10.08, 6.74), *p* = 0.69].	NA	Random-effects model	Critically low
15	Jianping et al. (56)	Inception to July 30, 2020.	13 (9 about liraglutide; 4 about exenatide)	MASLD (704)	RCT	Liraglutide: 0.9 mg, 1.2 mg,1.8 mg qd; Exenatide: 20μg bid	The standard care or placebo	12–48	56.25% (396)	39.0 ± 8.1–87.1 ± 3.3	26.8 ± 0.7–33.5 ± 5.5	(a) **Characteristics of liver:** HFC [MD = −1.4, 95% CI (−2.75, −0.05), *p* = 0.04]. (b) **Liver enzyme:** AST [MD = −3.04, 95% CI (−5.93, −0.16), *p* = 0.04]. (c) **Metabolic markers:** TG [MD = −0.2, 95% CI (−0.28, 0.13), *p* = 0.16], TC [MD = −0.12, 95% CI (−0.29, 0.05), *p* = 0.16], LDL-C [MD = −0.18, 95% CI (−0.36, 0.01), *p* = 0.06]. (d) **Anthropometric measurements:** BMI [MD = −1.15, 95% CI (−2.26, −0.04), *p* = 0.04], WC [MD = −3.87, 95% CI (−6.62, −1.12), *p* = 0.006].	Only mild AEts were reported. No serious AEs including severe hypotension, altered bowel habits or pancreatitis occurred were reported.	Random-effects model	Critically low
16	Wong et al. (57)	Inception to June 21, 2020	8 (3 about exenatide; 5 about liraglutide)	NAFLD combined with T2DM (1,452)	RCT, Retro-spective Study	Exenatide: Liraglutide:	The placebo or active agents group	NA	66.94 (972)	42.00 ± 3.20–56.40 ± 8.40	26.82 ± 3.66–30.59 ± 1.09	(a) **Characteristics of liver:** GLP-1 RAs significantly improved HFC [SMD = −1.05, 95% CI (−1.62, −0.48), *p* < 0.001, compared to baseline], [SMD = −0.54, 95% CI (−0.79, −0.29), *p* < 0.001, compared to the placebo]. (b) **Anthropometric measurements:** GLP-1RAs significantly reduced BMI [SMD = −0.98, 95% CI (−1.45, −0.51), *p* < 0.001], WC [SMD = −1.31, 95% CI (−2.47, −0.15), *p* = 0.03], hip circumference [SMD = −2.39, 95% CI (−3.06, −1.73), *p* < 0.001], waist-to-hip ratio [SMD = −0.69, 95% CI (−1.23, −0.16), *p* = 0.01], and VAT [SMD = −0.57, 95% CI (−0.94, −0.20), *p* < 0.01], compared to baseline; GLP-1RAs significantly reduced BMI [SMD = −1.01, 95% CI (−1.49, −0.52), *p* < 0.001], WC [SMD = −1.22, 95% CI (−2.22 to −0.22), *p* = 0.02], hip circumference [SMD = −3.71, 95% CI (−4.55, −2.87), *p* < 0.001], subcutaneous adipose tissue [SMD = −0.76, 95% CI (−1.21 to −0.32), *p* < 0.01]. and visceral adipose tissue [SMD = −0.66, 95% CI (−1.03 to −0.28), *p* < 0.01, compared to the placebo]. (c) **Liver enzyme:** GLP-1RAs significantly reduced total bilirubin [SMD = −5.83, 95% CI (−7.01, −4.66), *p* < 0.001], AST [SMD = −1.46, 95% CI (−2.22, −0.79), *p* < 0.001], ALT [SMD = −1.69, 95% CI (−2.32, −1.07), *p* < 0.001], and GGT [SMD = −2.10, 95% CI (−3.1 6, −1.04), *p* < 0.001]; GLP-1RAs significantly reduced APRI [SMD = −0.68, 95% CI (−1.24, −0.18), *p* = 0.02]; The AST/ALT ratio was significantly higher after GLP-1RAs treatment [SMD = 1.65, 95% CI (1.33, 1.97), *p* < 0.001]. All compared to baseline. (d) **Metabolic markers:** GLP-1RAs treatment resulted in a significant reduction in FBG [SMD = −2.03, 95% CI (−3.40, −0.65), *p* < 0.01), 2-h postprandial glucose [SMD = −2.15, 95% CI (−3.31, −0.98), *p* < 0.001], and HbA1c [SMD = −2.17, 95% CI (−3.39, −0.94), *p* < 0.01]; GLP-1RA treatment also significantly decreased HOMA-IR [SMD = −1.04, 95% CI (−1.35, −0.73), *p* < 0.001]; GLP-1RAs treatment also significantly reduced TC [SMD = −0.70, 95% CI (−1.38, −0.02), *p* = 0.04], TG [SMD = −0.84, 95% CI (−1.44, −0.24), *p* < 0.01], and FFA [SMD = −0.62, 95% CI (−1.10, −0.14), *p* = 0.01]; GLP-1RAs treatment significantly increased adiponectin [SMD = 0.84, 95% CI (0.58, 1.09), *p* < 0 .001]; GLP-1RAs significantly decreased CK-18 [SMD = −1.28, 95% CI (−1.70, −0.86), *p* < 0.001], IL-6 [SMD = −0.74, 95% CI (−1.33, −0.16), *p* = 0.01], and CRP [SMD = −2.02, 95% CI (−2.36, −1.68), *p* < 0.001]. All compared to baseline.	NA	Random-effects model	Critically low
17	Mantovani et al. (58)	Inception to December 15, 2020	11 (3 about exenatide; 1 about dulaglutide; 6 about liraglutide; 1 about semaglutide)	NAFLD/ NASH (935)	RCT	Exenatide: 5–10 μg bid/1.8 mg qd; Dulaglutide: 1.5 mg qw; Liraglutide: 1.8 mg qd/3.0 mg qd; Semaglutide: 0.1 mg qd.	The placebo or active agents group	12–72	51 (477)	49 ± 5	32 ± 3	(a) **Characteristics of liver:** Liraglutide or semaglutide was associated with a significantly greater histologic resolution of NASH with no worsening of liver fibrosis [RR 4.06, 95% CI (2.52, 6.55), Z-test = 5.74, *p* < 0.0001, *I^2^* = 0%]; GLP-1RAs have shown significantly improvement in liver fibrosis stage without worsening of NASH [RR 1.50, 95% CI (0.98, 2.28), Z-test = 1.86, *p* = 0.06, *I^2^* = 0%). (b) **Anthropometric measurements:** GLP-1RAs significantly reduced body weight [WMD = −4.06, 95% CI (−5.44, −2.68), Z-test = −5.76, *p* < 0.0001]. (c) **Liver enzyme:** GLP-1RAs significantly reduced ALT [WMD = −7.21, 95% CI (−13.35, −1.07), Z-test = −2.30, *p* = 0.02], and GGT [WMD = −10.97, 95% CI (−17.82, −4.12), Z-test = −3.14, *p* < 0.001]; AST levels did not differ between the two arms of treatment [WMD = −2.92, 95% CI (−8.15, 2.31), Z-test = −1.09, *p* = 0.27]. (d) **Metabolic markers:** GLP-1RAs significantly reduced HbA1c [WMD = −0.45%, 95% CI (−0.79, −0.12), Z-test = −2.65, *p* = 0.01].	Gastro-intestinal symptoms, such as nausea, constipation, diarrhea or abdominal discomfort.	Random-effects model	Critically low
18	Ghosal et al. (59)	NA	8 (1 about dulaglutide; 3 about exenatide; 3 about liraglutide; 1 about semaglutide)	NAFLD combined with T2DM (615)	RCT	Dulaglutide: 0.75–1.5 mg qw; Exenatide: 5–10 μg bid; Liraglutide: 0.6–1.8 mg; Semaglutide: 0.4 mg qd.	The placebo or active agents group	12–72	28.8 (177)	43 ± 4.1–60.7 ± 16.1	NA	(a) **Characteristics of liver:** GLP-1RAs significantly improved HFC [SDM = −0.43, 95% CI (−0.74, −0.12), *p* < 0.01]; biopsy resolution [RR = 6.60, 95% CI (2.67, 16.29), *p* < 0.01], compared to baseline. (b) **Anthropometric measurements:** GLP-1RAs significantly reduced body weight [SDM = −0.66, 95% CI (−0.88, −0.44), *p* < 0.01], compared to baseline. (c) **Liver enzyme:** GLP-1RAs significantly reduced ALT [SDM = −0.56, 95% CI (−0.88, −0.25), *p* < 0.01); AST [SDM = −0.44, 95% CI (−0.64, −0.24), *p* < 0.01]; GGT [SDM = −0.60, 95% CI (−0.86, −0.34), *p* < 0.01], compared to baseline. (d) **Metabolic markers:** GLP-1RAs significantly reduced HbA1c [SDM = −0.40, 95% CI (−0.61, −0.19), *p* < 0.01]; TG [SDM = −0.22, 95% CI (−0.42, −0.03), *p* = 0.02], compared to baseline.	NA	Random-effects model	Critically low
19	Kalogirou et al. (17)	Inception to May 2019	5 (All about liraglutide)	NAFLD/ NASH (371)	RCT	Liraglutide: 1.8 mg qd/3 mg qd/0.6–1.2 mg qd.	The placebo or active agents group	12–56	NA	NA	NA	(a) **Characteristics of liver:** Liraglutide induced a non-significant decrease in HFC by 3.63% [95% CI (−9.59, 2.33), *I^2^* = 93%]. (b) **Anthropometric measurements:** Liraglutide reduced in BMI by 0.71 kg/m^2^ [95% CI (−1.20, −0.22), *I^2^*= 79%]. (c) **Liver enzyme:** Liraglutide induced a non-significant decrease in ALT by 5.66 U/L [95% CI (−11.53, 0.21), *I^2^* = 67%]. (d) **Metabolic markers:** Liraglutide did not affect significantly the corresponding levels of TC [MD = 0.10, 95% CI (−0.01, 0.22), *I^2^* = 17%], LDL-C [MD = 0.09, 95% CI (−0.15, 0.32), *I^2^*= 74%], and HDL-C [MD = 0.05, 95% CI (−0.02, 0.12), *I^2^* = 54%]; Liraglutide induced a non-significant decrease in HOMA-IR levels by 0.11 [95% CI (−1.17, 0.95), *I^2^* = 39%].	Gastrointestinal disorder	Random-effects model	Critically low
20	Mantovani et al. (60)	Inception to March 1, 2020	7 (3 about exenatide; 4 about liraglutide)	NAFLD/ NASH (472)	RCT	Exenatide: 5–10 μg bid/ 1.8 mg qd; Liraglutide: 1.8 mg qd.	The placebo or active agents group	12–24	66 (311)	47 ± 3	32 ± 3	(a) **Characteristics of liver:** GLP-1 RAs significantly improved HFC [WMD = −6.23%, 95% CI (−8.95%, −3.51%)]; 40% of patients' histological resolution of NASH with no worsening of fibrosis (40% vs. 9%; *p* < 0.05, in favor of GLP-1 RAs). (b) **Anthropometric measurements:** GLP-1 RAs also improved intra-abdominal visceral adipose tissue [WMD = −27.43 cm^2^, 95% CI (−42.66, −12.2)]. (c) **Liver enzyme:** GLP-1 RAs decreased the levels of ALT [WMD = −8.77, 95% CI (−17.69, 0.14)], GGT [WMD = −10.17, 95% CI (−14.27, −6.07)], and AST [WMD = −3.41, 95% CI (−11.49, 4.68)]. (d) **Metabolic markers:** GLP-1 RAs decreased the levels of HbA1c [WMD = −0.13, 95% CI (−0.47%, 0.21%)].	NA	Random-effects model	Critically low
21	Teshome et al. (61)	Inception to February 14, 2018	10 (3 about exenatide; 5 bout liraglutide; 2 about exenatide and liraglutide)	NAFLD/ NASH/ fibrosis (590)	RCT; Cohort; Single arm trial; Single centered trial.	Exenatide: 5–10 μg bid; Liraglutide: 0.3–0.9 mg qd/0.6–1.2 mg qd/1.8 mg qd/0.6–3 mg qd; Sitagliptin: 50 mg bid.	The placebo or active agents group	12–96	NA	NA	NA	(a) **Liraglutide:** A significant reduction of ALT, AST, GGT, and HFC; Improvement of inflammation; liraglutide (1.2 mg qd) showed a significant reduction of body weight; Liraglutide (0.9 mg qd) for 48 weeks showed no significant change was seen in body weight; (b) **Exenatide:** A significant reduction of ALT, AST, and GGT levels (vs. metformin and insulin therapy); Improvement of steatosis, body weight.	Gastrointestinal discomfort	NA	Critically low
22	Fan et al. (31)	Inception to October 2019	8 (2 about exenatide; 6 about liraglutide)	NAFLD (406)	RCT	Liraglutide: max 1.8 mg/max 3 mg; Exenatide max 20 ug.	The placebo or active agents group	12–48	48 (29)−92 (22)	41–51	27.9–35.9	(a) **Characteristics of liver:** Significant overall effects of GLP-1RAs on LFF [SMD = −0.33, 95% CI (−0.64, −0.03), *p* = 0.034, *I^2^* = 0%]; A resolution of NASH (defined as the disappearance of hepatocyte ballooning without worsened fibrosis) [RR = 4.5, 95% CI (1.1, 18.9), *p* = 0.02, in favor of liraglutide); Fewer fibrosis progression [RR = 0.2, 95% CI (0.1, 1.0), *p* = 0.04, in favor of liraglutide]; Liraglutide has shown improvements in hepatocyte ballooning [RR = 1.9, 95% CI (1.0, 3.8), *p* = 0.05] and steatosis [RR = 1.8, 95% CI (1.1, 3.0), *p* = 0.009]. (b) **Anthropometric measurements:** A significant effect of GLP-1RAs on BMI [SMD = −0.89, 95% CI (−1.60, −0.19), *p* = 0.012]. (c) **Liver enzyme:** A significant difference on ALT [SMD = −1.25, 95% CI (−1.68, −0.82), *p* = 0.000, *I^2^*= 37.5%, *p* = 0.206] (exenatide vs. control); No significant effect on ALT (GLP-1RAs vs. control; liraglutide vs. control); A significant difference on AST [SMD = −0.62, 95% CI (−1.16, −0.08), *p* = 0.024, *I^2^* = 64%, *p* = 0.096] (exenatide vs. control); No significant effect on AST (GLP-1RAs vs. control; liraglutide vs. control). (d) **Metabolic markers:** A significant effect on adiponectin [SMD = 0.66, 95% CI (0.37, 0.95), *p* = 0.000**]**; No significant difference in the GLP-1RAs for TC [SMD = 0.00, 95% CI (−0.21, 0.21), *p* = 0.990), TG [SMD = −0.12, 95% CI (−0.33, 0.09), *p* = 0.261], HDL-L [SMD = 0.10, 95% CI (−0.12, 0.33), *p* = 0.370], and LDL-L [SMD = 0.05, 95% CI (−0.17, 0.28), *p* = 0.650].	NA	Random- effects model; Fixed-effects model	Low
23	Lv et al. (62)	Inception to August 23, 2020	9 (4 about exenatide; 5 about liraglutide)	T2DM combined with NAFLD (780)	RCT	Exenatide; Liraglutide.	The placebo or active agents group	12–26	NA	40–56	NA	(a) **Anthropometric measurements:** Body weight [WMD = −4.20, 95% CI (−8.15, −0.25), *p* = 0.03, *I^2^*= 97.6%]; BMI [WMD = −1.57, 95% CI (−2.74, – 0.39), *p* = 0.009, *I^2^* = 97.8%]; WC [WMD = −4.14, 95% CI (−7.09, −1.19), *p* = 0.006, *I^2^*= 94.7%]; WHR [WMD = −0.01, 95% CI (−0.03, 0.02), *p* = 0.57, *I^2^* = 96.2%]. (b) **Metabolic markers:** PPG [WMD = −25.73 mg/dl, 95% CI (−32.71, −18.75), *p* < 0.001, *I^2^* = 11.9%]; FBS [WMD = −2.12 mg/dl, 95% CI (−6.23, 1.96), *p* = 0.31, *I^2^*= 15.8%]; HbA1c [WMD = −0.08%, 95% CI (−0.21, 0.04), *p* = 0.18, *I^2^* = 0.0%]; HOMA-IR [WMD = −0.31, 95% CI (−0.69, 0.07), *p* = 0.11, *I^2^* = 92.9%].	NA	Random-effects model	Low
24	Lv et al. (63)	Inception to November 2, 2019	24 (12 about liraglutide; 10 about exenatide; 2 about dulaglutide; 1 about semaglutie)	NAFLD (6,313)	RCT; Retro-spective studies; Case series	Liraglutide: 0.6 mg, 0.9 mg, 1.2 mg,1.8 mg, 3 mg qd; Exenatide: 5–10 μg bid; Dulaglutide: 0.75 mg, 1.5 mg qw; Semaglutie: 0.05–0.4 mg qd.	The standard care or placebo	12–144	NA	NA	NA	(a) **Characteristics of liver:** 6 out of 8 studies demonstrated a significant reduction in HFC with GLP-1 RA therapy; 3 out of 4 studies showed significant improvement in the magnitude of liver fibrosis with GLP-1 RA therapy. (b) **Liver enzyme:** 19 out of 21 studies supported the efficacy of GLP-1 RAs on the improvement in hepatic enzymes (ALT, AST, and GGT).	NA	NA	Critically low
25	Dai et al. (12)	Inception to April 20, 2020	8 (6 about exenatide; 2 about liraglutide)	MASLD (396)	RCT	Exenatide: 10 ug bid; Liraglutide: 1.2 mg qd/1.8 mg qd/3 mg qd.	The placebo or active agents group	12–48	NA	40.7 ± 9.1–51.0 ± 11.4	NA	**The primary outcome:** (a) **The reduced severity of MASLD:** The resolution of definite non-alcoholic steatohepatitis (39% vs. 2%, liraglutide vs. placebo); Fewer patients in the liraglutide group exhibited progression of fibrosis and a greater improvement in steatosis and hepatocyte ballooning compared with placebo group. (b) **The reduction in HFC:** GLP-RAs significantly improved the HFC [WMD = −3.17%, 95% CI (−5.30, −1.03), *p* < 0.0001, *I^2^* = 31%]. **The secondary outcomes:** (a) **Anthropometric measurements:** Both liraglutide and exenatide therapy significantly reduced body weight [WMD = −3.25 kg 95% CI (−6.73, −0.74), *p* = 0.03 vs. WMD = −7.40 kg, 95% CI (−14.55, −0.26), *p* = 0.04] and WC [WMD = −2.61 cm, 95% CI (−5.35, 0.13), *p* = 0.06 vs. WMD = −6.74 cm, 95% CI (−11.11, −2.36), *p* = 0.003]. (b) **Liver enzyme:** The GLP-RAs improved ALT [WMD = −10.73 U/L, 95% CI (−20.94, −0.52), *p* = 0.04, *I^2^* = 74%]; Subgroup analysis the different therapies revealed a significant difference in the reduction of ALT in the exenatide group but not in liraglutide [WMD = −22.16 U/L, 95% CI (−38.44, −5.88), *p* = 0.008, *I^2^* = 84% vs. WMD = −5.21 U/L, 95% CI (−12.93, 2.51), *p* = 0.19]. Non-significant different of the GLP-RAs in AST [WMD = −0.17 U/L, 95% CI (−0.44, 0.09), *p* = 0.15, *I^2^* = 29%]. A significant effect of the GLP-RAs on γ-GGT [WMD = −12.25 U/L, 95% CI (−18.85, −5.66), *p* = 0.0003, *I^2^* = 23%]. **Metabolic markers:** A significant difference of the GLP-RAs on FBG [WMD = −0.36 mmol/L, 95% CI (−0.69, −0.03), *p* = 0.030, *I^2^* = 39%] and a significant reduction in Hb1Ac [WMD = −0.36%, 95% CI (−0.52, −0.19), *p* < 0.0001, *I^2^* = 0%].	Gastrointestinal complications (involving nausea, vomiting, diarrhea, decreased appetite, flatulence, and abdominal pain) with no serious adverse events.	Random-effects model	Critically low
26	Tang et al. (64)	Inception to March 2016	4 (2 about liraglutide; 2 about exenatide)	NAFLD combined with T2DM (141)	RCT	Liraglutide: 1.8 mg qd; Exenatide: 10 μg bid	The standard care or placebo	12–50	NA	NA	NA	(a) **Liver enzyme:** ALT [MD = −8. 36, 95% CI (−13. 41, −3. 31), *p* = 0.001]. (b) **Metabolic markers:** HbA1c [MD = −0.43, 95% CI (−0.73, −0.13), *p* = 0.005], FBG [MD = −0.71, 95% CI (−1.39, −0.03), *p* = 0.04], TG [MD = −0.49, 95% CI (−0.82, −0.16), *p* = 0.004], LDL-C [MD = −0.12, 95% CI (−0.45, −0.21), *p* = 0.04], and TC [MD = −0.15, 95% CI (−0.46, 0.12), *p* = 0.35]. (c) **Anthropometric measurements:** BMI [MD = −1.83, 95% CI (−2.18, −0.58), *p* = 0.0008].	NA	Fixed-effects model	
27	Dong et al. (65)	MEDLINE: January 1966 to February 2016); Embase: January 1974 to February 2016.	6 (3 about exenatide; 3 about liraglutide)	NAFLD/ NASH (329)	RCT, observa-tion cohort study	Exenatide: 10 ug bid; Liraglutide: 0.9 mg qd/1.8 mg qd.	The placebo or active agents group	12–144	NA	NA		(a) **Characteristics of liver:** GLP-1RA treatment showed significant reductions in scores compared with baseline for steatosis [MD = 0.80, 95% CI (0.49, 1.11), lobular inflammation [MD = 0.22, 95% CI (0.00, 0.45), hepatocellular ballooning [MD = 0.41, 95% CI (0.15 to 0.67)] and fibrosis [MD = 0.35, 95% CI (0.00, 0.70)]; And significant improvement rates were observed for steatosis [OR 0.71, 95% CI (0.50, 0.86)], lobular inflammation [OR 0.42, 95% CI (0.28, 0.57)], hepatocellular ballooning [OR 0.56, 95% CI (0.41, 0.70), and fibrosis [OR 0.42, 95% CI (0.23, 0.65)]. (b) **Anthropometric measurements:** All studies have shown a significant weight loss in NAFLD/NASH patients. (c) **Liver enzyme:** A significant reduction on GGT [MD = 13.8, 95% CI (7.4, 20.3), *p* < 0.001]; Substantial heterogeneity in the estimates of the effects of GLP-1RAs on ALT and AST levels (*I^2^* = 77% and 76%, results not shown). (d) **Metabolic markers:** Significant mean decreases of FBG (range from 0.9 to 2.84 mmol/L); 2-h postprandial plasma glucose (2.59 to 5.49 mmol/L), and HbA1c (0.48 to 1.42%).	Gastrointestinal discomfort, including nausea, vomiting, decreased appetite, abdominal pain, and diarrhea. Adverse events tended to occur more frequently with higher doses.	Random- effects model	Critically low

WMD, Weighted mean differences; MD, Mean differences; SMD, Standardized mean difference; RR, Risk ratio; OR, Odds ratio; 95 % Cis, 95 % confidence intervals; FFA, Free fatty acids; HDL, High density lipoprotein; LDL, Low density lipoprotein; HbA1c, Glycated hemoglobin A1c; FBG, Fasting Blood Glucose; CRP, C-reactive protein; IL-6, Interleukin-6; CK-18, Cytokeratin-18; HFC, Hepatic Fat Content; BMI, Body mass index; AST, Aspartate aminotransferase; ALT, Alanine aminotransferase; GGT, Gamma-glutamyl transferase; ALP, Alkaline phosphatase; VAT, Visceral adipose tissue; SAT, Subcutaneous adipose tissue; INH, Intrahepatic adipose; NAS, NAFLD Activity Score; MRI-PDFF, MRI proton density fat fraction; ELFTS, Enhanced Liver Fibrosis Test Score.

### 3.3 GLP-1 RAs and MASLD

Twenty-four meta-analyses primarily examined the efficacy of GLP-1 RAs in MASLD from the five aspects mentioned above. Three meta-analyses briefly discussed AEs associated with GLP-1 RAs in patients with MASLD. One meta-analysis focused primarily on AEs.

#### 3.3.1 Efficacy of GLP-1 RAs on liver characteristics in MASLD

**Figure 2:**
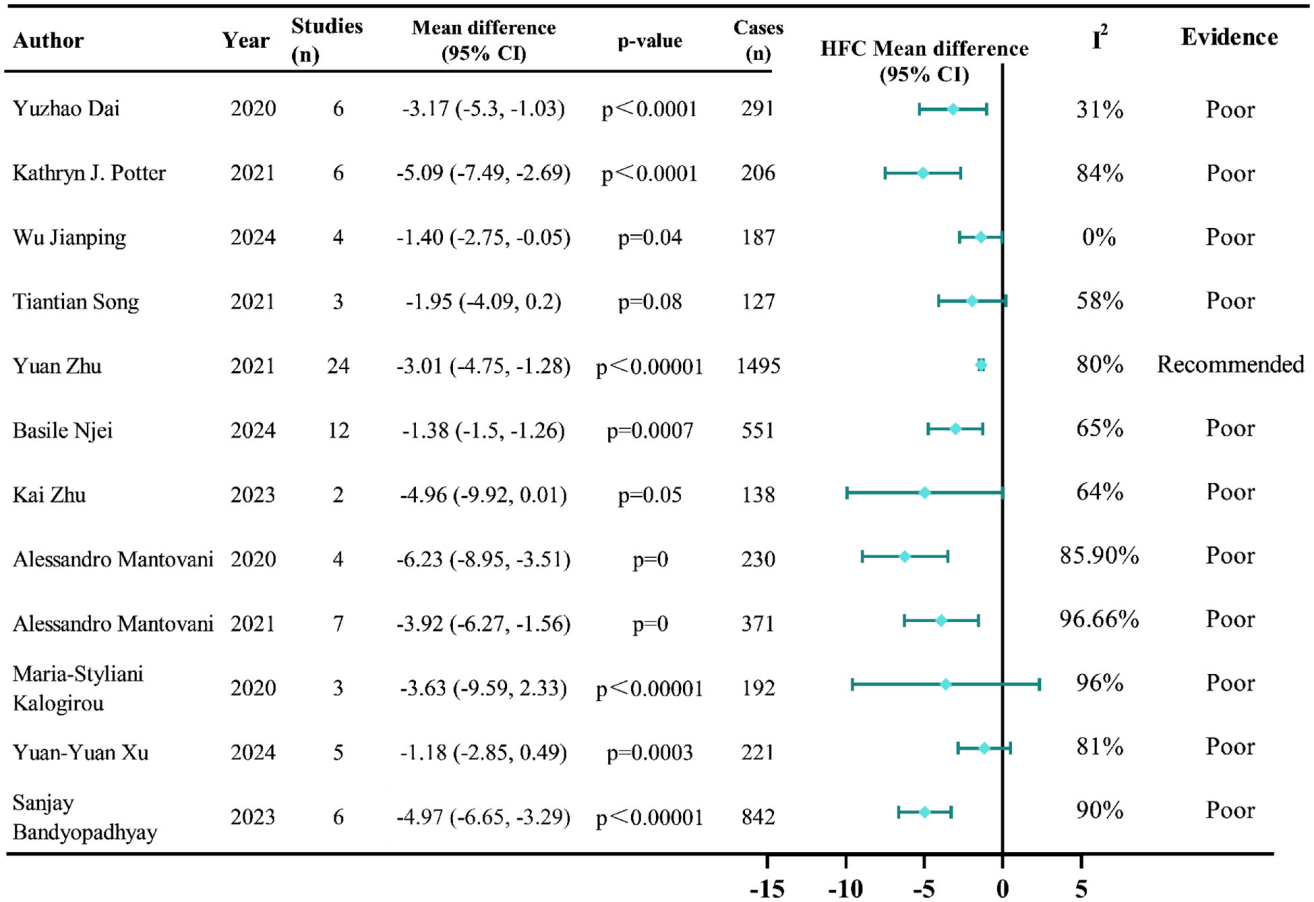
Pooled estimation of the effect of GLP-1 RAs on HFC in MASLD.

Among the 24 meta-analyses, 12 reviews investigated the effect of GLP-1 RAs on HFC in MASLD, four examined histologic resolution, and one studied FIB-4 (Supplementary Tables S5, S6). These studies reported that most kinds of GLP-1 RAs can improve the liver characteristics in MASLD. All 12 reviews observed a regression from a greater to a lower degree of HFC with MD ranging from −1.18 to −6.23, with 58.33% of them showed significant improvement. All four reviews revealed that treatment with GLP-1 RAs was associated with a significantly greater histologic resolution [the pooled random-effects odds ratio (OR) ranged from 3.18 to 4.45]. However, no significant resolution of the FIB-4 was observed. One of these findings was classified as recommended evidence, whereas the rest were classified as poor because they were based on small-sample studies (*n* < 1,000). The pooled estimates derived from the included reviews are depicted in Figure 2 and Supplementary Figure S4.

**Figure 3:**
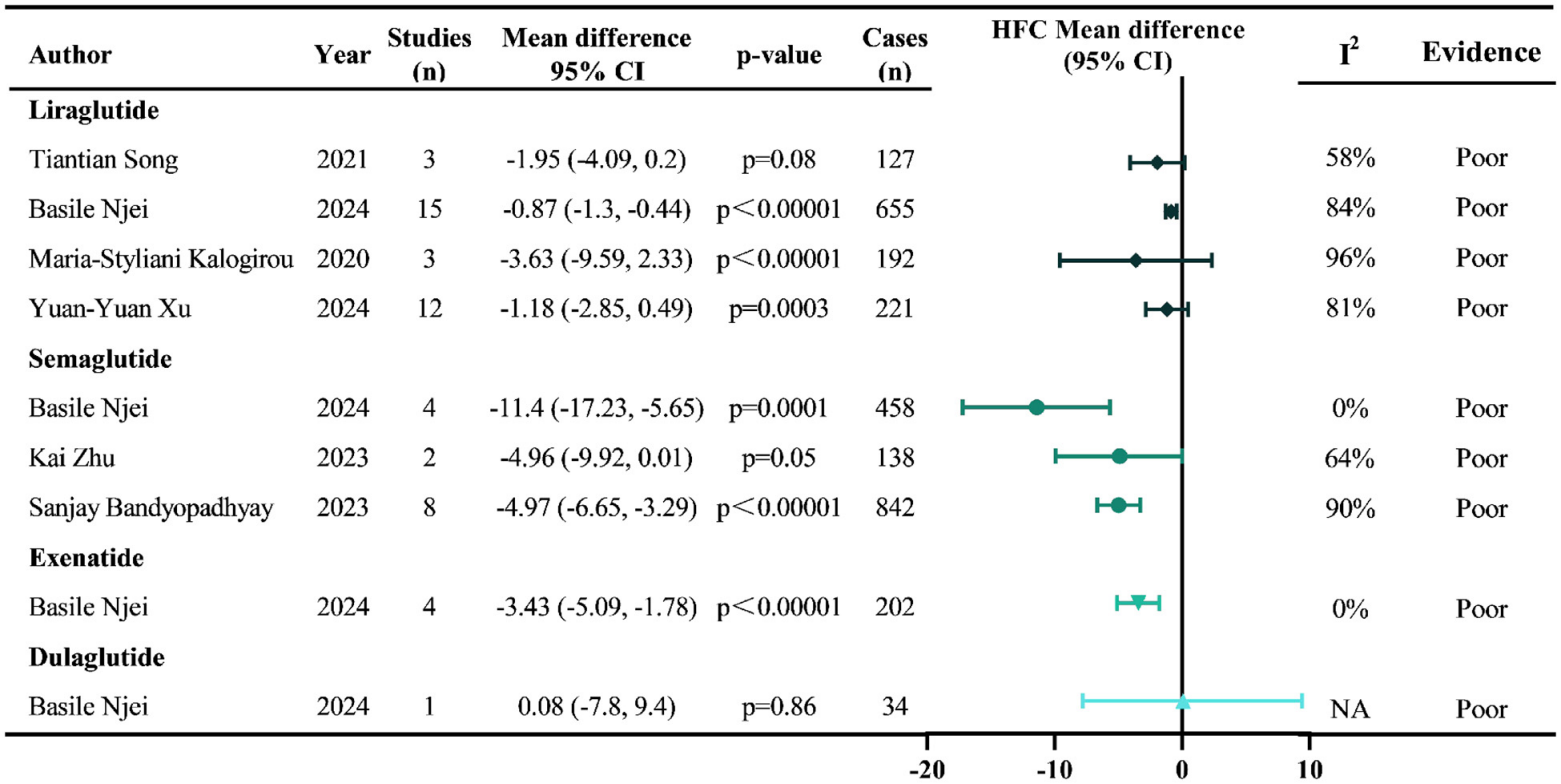
Pooled estimation of the effect on HFC of liraglutide, semaglutide, exenatide, and dulaglutide in MASLD.

Four meta-analyses investigated the effects of liraglutide on MASLD, observing an improvement in HFC with MD from −0.87 to −3.63. Three reviews examined the effect of semaglutide on MASLD, most indicating a significant reduction in HFC with MD from −4.96 to −11.4. Only two reviews assessed the efficacy of exenatide and dulaglutide separately, revealing a significant improvement in HFC with exenatide treatment [MD = −3.45, 95% CI (−5.09, −1.78), *p* < 0.00001]; however, no significant reduction was observed dulaglutide [MD = 0.08, 95% CI (−7.8, 9.4), *p* = 0.86]. Among four reviews on the effect on histologic resolution, one investigated only liraglutide, showing significant histologic resolution in MASLD with OR = 3.18. However, no review has separately studied the other four types. One review examined the effect of liraglutide using subgroup analysis according to the population with or without type 2 diabetes mellitus (T2DM), which did not show a significant reduction in HFC [for patients with T2DM: MD = −5.14, 95% CI (−11.93, 1.66); *I*^2^ = 96%] or for patients without T2DM [MD = 1.10, 95% CI (−6.49, 8.69); *I*^2^ = not applicable]. The pooled estimates derived from the reviews are shown in Figure 3.

#### 3.3.2 Efficacy of GLP-1 RAs on liver enzymes in MASLD

**Figure 4:**
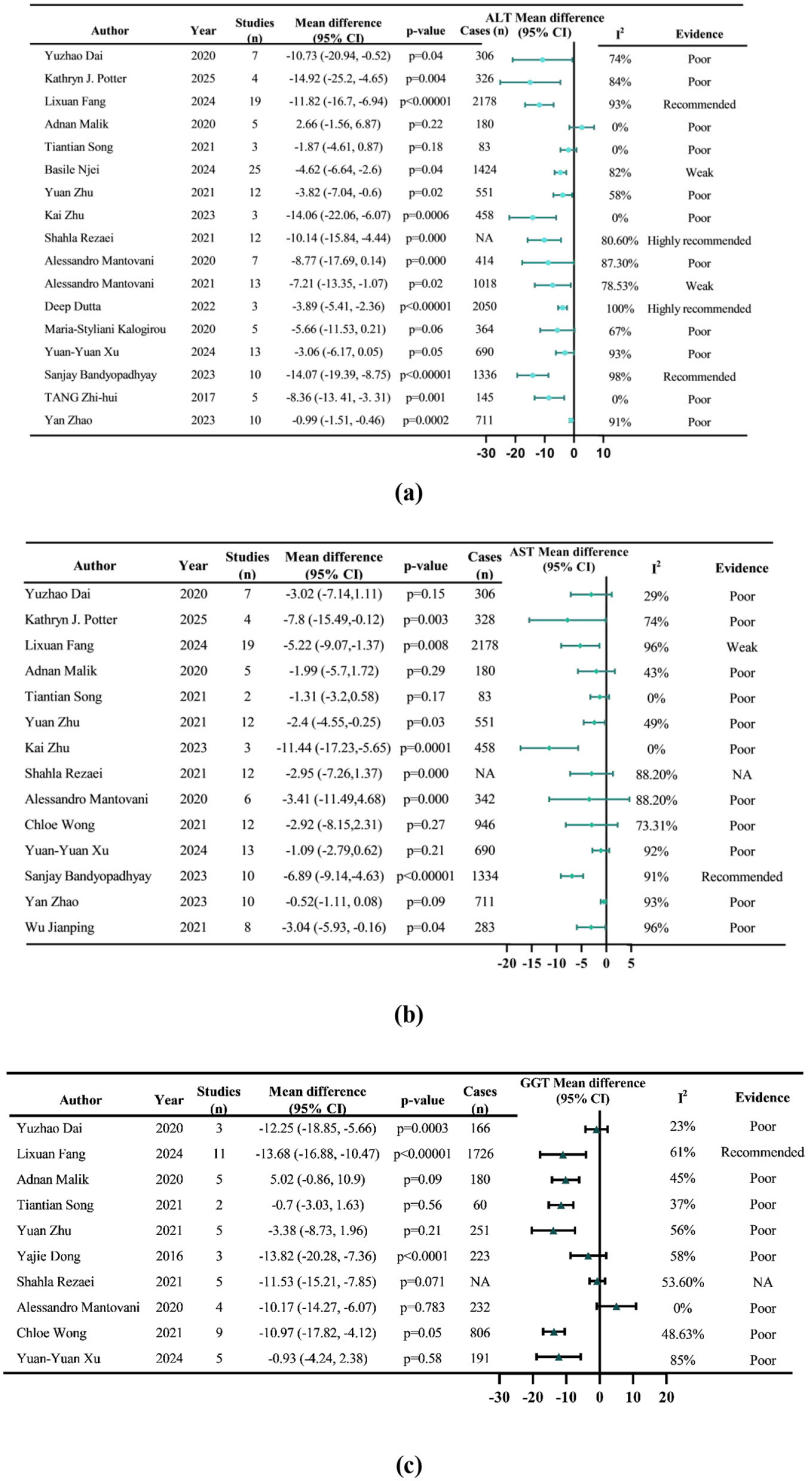
Pooled estimation of the effect of GLP-1 RAs on liver enzymes level in MASLD, including ALT **(a)**, AST **(b)**, GGT **(c)**.

Twenty reviews of the 24 meta-analyses examined the efficacy of GLP-1 RAs on liver enzymes, including ALT, AST, GGT, and ALP (Supplementary Table S6). The pooled estimates derived from the included reviews are shown in Figure 4. Most studies observed a significant reduction in liver enzyme levels, and semaglutide demonstrates advantages in reducing liver enzymes. Eighty-five percentage of the 20 studies, investigated the influence of GLP-1 RAs on ALT levels in MASLD. When compared to placebo or reference therapy, GLP-1 RAs decreased the levels of ALT with MD from −14.92 to 2.66, and 70.59% of them reported a significant decrease in ALT. Seventy percentage reviews investigated the effect of GLP-1 RAs on AST, and a significant reduction in AST was observed in six meta-analyses with MD from −11.44 to −0.52. Fifty percentage reviews explored the effect of GLP-1 RAs on GGT levels with MD of reduced GGT levels from −13.82 to 5.02. Two results were classified as highly recommended evidence, four as recommended evidence, and the remaining as weak evidence.

Six reviews investigated the effect of liraglutide on ALT, four reviews investigated semaglutide, and two investigated exenatide and dulaglutide separately. Significant decreases in ALT levels were observed when semaglutide was compared with placebo or reference therapy with MD from −14.06 to −3.89. However, no significant reduction in ALT levels was observed after treatment with liraglutide, exenatide, or dulaglutide. Three reviews investigated the effect of liraglutide on AST, while two reviews examined the effect of semaglutide. Semaglutide treatment significantly reduced AST levels with MD from −11.44 to −6.89; however, no significant reduction was observed with liraglutide (Supplementary Figure S5).

#### 3.3.3 Efficacy of GLP-1 RAs on lipid metabolism in MASLD

**Figure 5:**
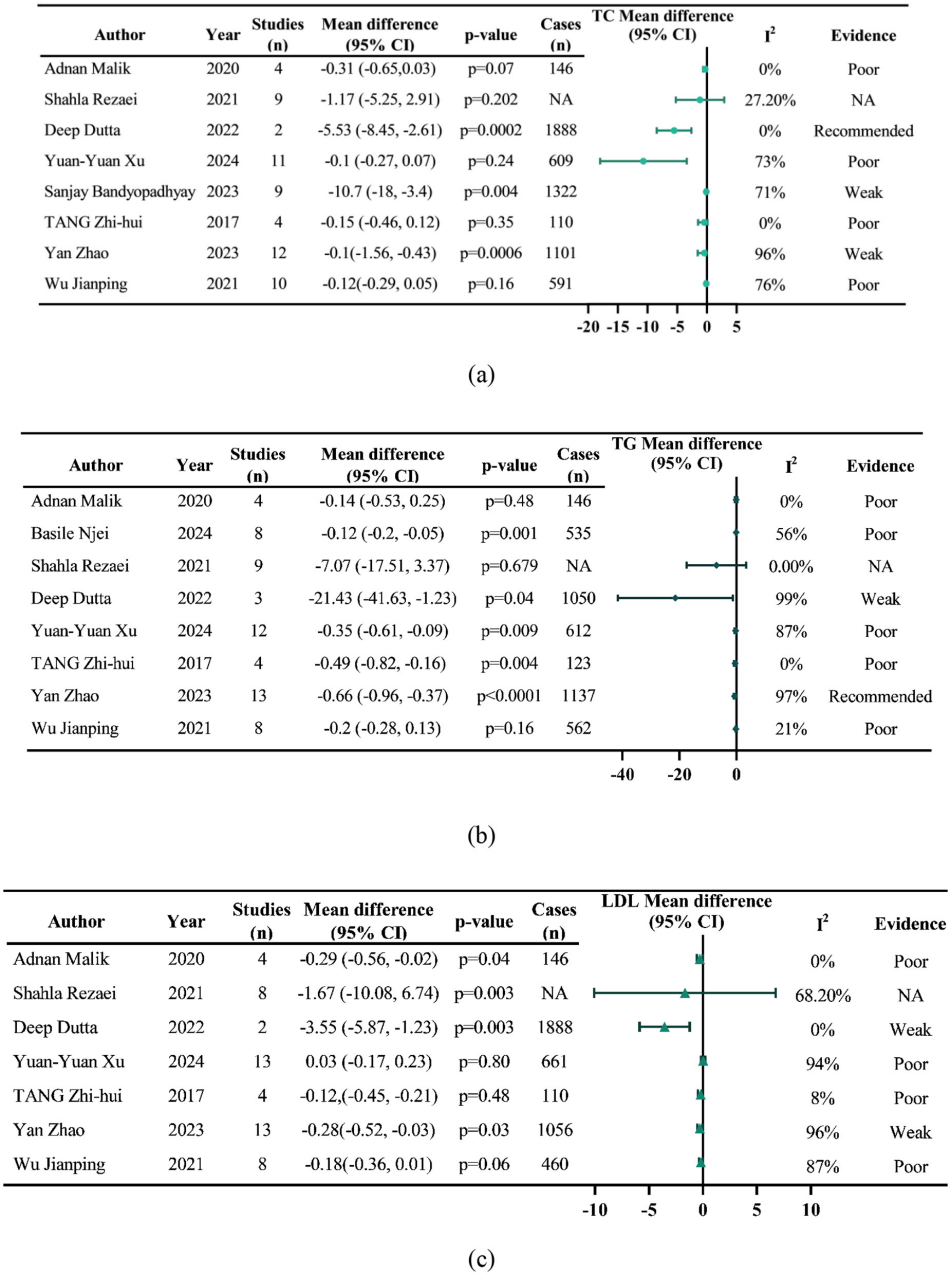
Pooled estimation of the effect of GLP-1 RAs on blood lipid level in MASLD, including TC **(a)**, TG **(b)**, and LDL **(c)**.

Out of the 25 meta-analyses, eight reviews investigated the effect of GLP-1 RAs on lipid metabolism. The pooled estimates derived from the included reviews are shown in Figure 5. Most of them found that GLP-1 RAs, especially semaglutide can reduce the level of lipid in MASLD. Eight reviews explored the effect on Total Cholesterol (TC); however, only 37.5% of them reported significant reduction of TC with MD from −10.7 to −0.1 (28–30). Eight reviews examined Triglyceride (TG) levels, 62.5% of them reported significant decreases with MD of TG from −0.12 to −21.43 (13, 16, 30–32). Seven meta-analyses investigated the effect on LDL, 57.14% of them observed significant reduction with MD from −3.55 to −0.29 (14, 28, 30, 32). Two were classified as recommended evidence, five as weak evidence, and 13 as poor evidence (Supplementary Table S6).

When classified by different types of GLP-1 RAs, significant resolution was observed in the reviews of semaglutide. Most reviews on liraglutide showed non-significant resolution of lipid markers. No significant decrease was observed in the studies on exenatide and dulaglutide (Supplementary Figure S6).

#### 3.3.4 Efficacy of GLP-1 RAs on glucose metabolism in MASLD

Nine reviews examined glucose metabolism. The pooled estimates derived from the included reviews are shown in Supplementary Figure S7. Studies included in this review showed that GLP-1 RAs can improve the glucose level in MASLD. Eight of these reviews reported a significant decrease in HbAc1. Only four reviews investigated the effect on FBG, three of which showed a significant decrease with MD from −0.36 to −1.35. One meta-analysis investigated the effect of GLP-1 RAs on Homeostatic Model Assessment for Insulin Resistance; however, no significant resolution was observed. Among this evidence, four were classified as recommended and seven as poor (Supplementary Table S6).

#### 3.3.5 Other efficacy of GLP-1 RAs in MASLD

Anthropometric measurements, including body weight, BMI, WC, SAT, and VAT, as well as inflammatory factors, such as CRP, were also included in the meta-analyses (Supplementary Table S6). The findings suggested that GLP-1 RAs can improve the anthropometric measurements of MASLD. Six reviews explored the effect of GLP-1 RAs on body weight, with five reporting a significant decrease. Eight reviews investigated the efficacy of GLP-1 RAs on BMI, all of which reported significant resolution. Four reviews examined the effect of GLP-1 RAs on WC, three of which reported a significant decrease. Two reviews investigated the influence of GLP-1 RAs on VAT and SAT, with both showing significant resolution. Pooled estimates derived from the included reviews are shown in Supplementary Figures S8, S9. One review studied the effect on CRP [MD = −0.41, 95% CI (−0.78, −0.04), *p* = 0.03].

#### 3.3.6 Safety of GLP-1 RAs in MASLD

Four meta-analyses investigated the safety of GLP-1 RAs (28, 30, 33, 34). Only one review discussed the AEs of GLP-1 RAs on MASLD (34). Patients undergoing GLP-1RA treatment had an overall higher incidence of AEs than the control group [OR 2.40, 95% (1.10, 5.26), *p* = 0.03]; however, no significant findings were observed in other meta-analyses (28). Additionally, when the treatment follow-up period was < 30 weeks, GLP-1 RA treatment was associated with a higher incidence of AEs than in the control group; however, this was not observed when the treatment follow-up period exceeded 30 weeks [OR 3.58, 95% CI (1.75, 7.32), *p* = 0.0005 vs. OR 1.74, 95% CI (0.55, 5.52), *p* = 0.35]. The incidence of serious AEs was discussed in three meta-analyses; however, no significant differences were observed. One review also evaluated the incidence rate of mild to moderate AEs, observing no significant resolution [OR 1.29, 95% CI (0.92, 1.80), *p* = 0.14] (34). In addition, gastrointestinal AEs were observed [OR 4.83, 95% CI (3.36, 6.95), *p* < 0.00001] in the treatment of MASLD with GLP-1 RAs, with an overall mean incidence rate of 58.9%, among which nausea was the most frequently reported (32.54%) (34). This significant resolution was also observed in another meta-analysis [OR 3.72, 95% CI (1.68, 8.23), *p* = 0.001] (33). One meta-analysis investigated the incidence of diarrhea, nausea, vomiting, decreased appetite, and constipation in the treatment of MASLD with semaglutide, all of which had a significant effect (28).

According to the epidemiological evidence assessment, two findings were classified as recommended evidence, one as weak evidence, and 10 as poor evidence (Table 2).

**Table 2:** Pooled estimation and epidemiological credibility assessment of the included meta-analyses on the safety of GLP-1 RAs in MASLD.

No.	Author	Year	Studies (*n*)	Outcome	Odds ratio (95% CI)	*p-value*	Cases (*n*)	*I^2^*	Evidence
1	Deep Dutta	2022	4	Adverse events	2.13 (0.76, 7.96)	*p* = 0.14	2,115	82%	Poor
2	Xiaoyan Huang	2025	10	Adverse events	2.4 (1.1, 5.26)	*p* = 0.03	960	70%	Poor
3	Yan Zhao	2023	7	Adverse events	2.53 (1.57, 4.07)	*p* = 0.0001	571	9%	Poor
1	Kai Zhu	2023	3	Serious adverse events	1.4 (0.75, 2.62)	*p* = 0.29	456	0%	Poor
2	Deep Dutta	2022	4	Serious adverse events	1.07 (0.69, 1.65)	*p* = 0.77	2,115	33%	Poor
3	Xiaoyan Huang	2025	6	Serious adverse events	1.46 (0.81, 2.63)	*p* = 0.21	704	0%	Poor
1	Xiaoyan Huang	2025	8	Mild to moderate adverse events	1.29 (0.92, 1.80)	*p* = 0.14	545	63%	Poor
1	Kai Zhu	2023	2	Gastrointestinal related side effects	3.72 (1.68, 8.23)	*p* = 0.001	390	49%	Poor
2	Xiaoyan Huang	2025	8	Gastrointestinal related side effects	4.83 (3.36, 6.95)	*p* < 0.00001	766	40%	Poor
1	Deep Dutta	2022	4	Diarrhea	2.05 (1.17, 3.6)	*p* = 0.01	2,115	66%	Weak
1	Deep Dutta	2022	4	Nausea	4.98 (3.23, 7.67)	*p* < 0.00001	2,115	0%	Recommended
1	Deep Dutta	2022	4	Vomiting	3.9 (1.75, 8.68)	*p* = 0.0009	2,115	54%	Recommended
1	Deep Dutta	2022	3	Decreased appetite	5.25 (2.68, 10.29)	*p* < 0.00001	465	0%	Poor
1	Deep Dutta	2022	3	Constipation	3.79 (1.63, 8.78)	*p* = 0.002	465	46%	Poor

### 3.7 Quality assessment

Five (21.7%) studies were rated as low quality, and the rest were rated as critically low quality (Supplementary Table S7). The primary limitations identified included the absence of a list of excluded studies and justification, as well as a written or registered protocol for the included studies, a lack of detailed funding sources, an assessment of risk of bias, and a discussion regarding the heterogeneity observed.

## 4 Discussion

### 4.1 Main findings

Twenty-three systematic reviews and meta-analyses of GLP-1 RAs in MASLD, published between 2016 and 2025, with 51 distinct pooled analyses, were analyzed for their efficacy from five aspects. The estimates of the efficacies of different GLP-1 RAs were also pooled. All GLP-1 RAs safely improved MASLD from these five aspects without fatal AEs such as severe hypoglycemia and pancreatitis. Furthermore, semaglutide showed outstanding efficacy.

Nearly half of the reviews explored the effect of GLP-1 RAs on HFC in MASLD, and significant regressions in HFC were observed in 63.63% of studies. Although few meta-analyses have investigated the efficacy of GLP-1 RAs on the histologic resolution of MASLD, all have shown significant improvements. NHS and FIB-4 showed non-significant resolution, which may be attributed to the limited number of reports. Additionally, evidence from the meta-analyses indicated that treatment with GLP-1 RAs resulted in the resolution of liver enzymes; although a decrease in ALT and AST levels was observed with liraglutide, only one of these five showed significant differences. Lipid metabolism and TC, TG, and LDL levels improved in MASLD; however, some studies showed no significant improvement. These insignificant changes may be attributed to bias caused by the small sample size of the included studies. Furthermore, semaglutide treatment significantly reduced the levels of liver enzymes and lipid indicators. A non-significant decrease in FBG was observed, which may be because FBG represents the blood glucose level at a specific point, which is influenced by various factors. However, HbA1c, an indicator reflecting glucose levels over the past 3 months, showed a significant improvement in 87.5% of studies. Additionally, treatment with GLP-1 RAs in MASLD resulted in significant improvement in body weight, BMI, VAT, and SAT.

All meta-analyses observed AEs in the GLP-1 RAs group. However, GLP-1 RAs demonstrated good safety, and no fatal AEs were reported. The incidence of serious AEs was not significant, and the most frequently reported AEs were associated with the gastrointestinal system.

### 4.2 Underlying mechanisms

**Figure 6:**
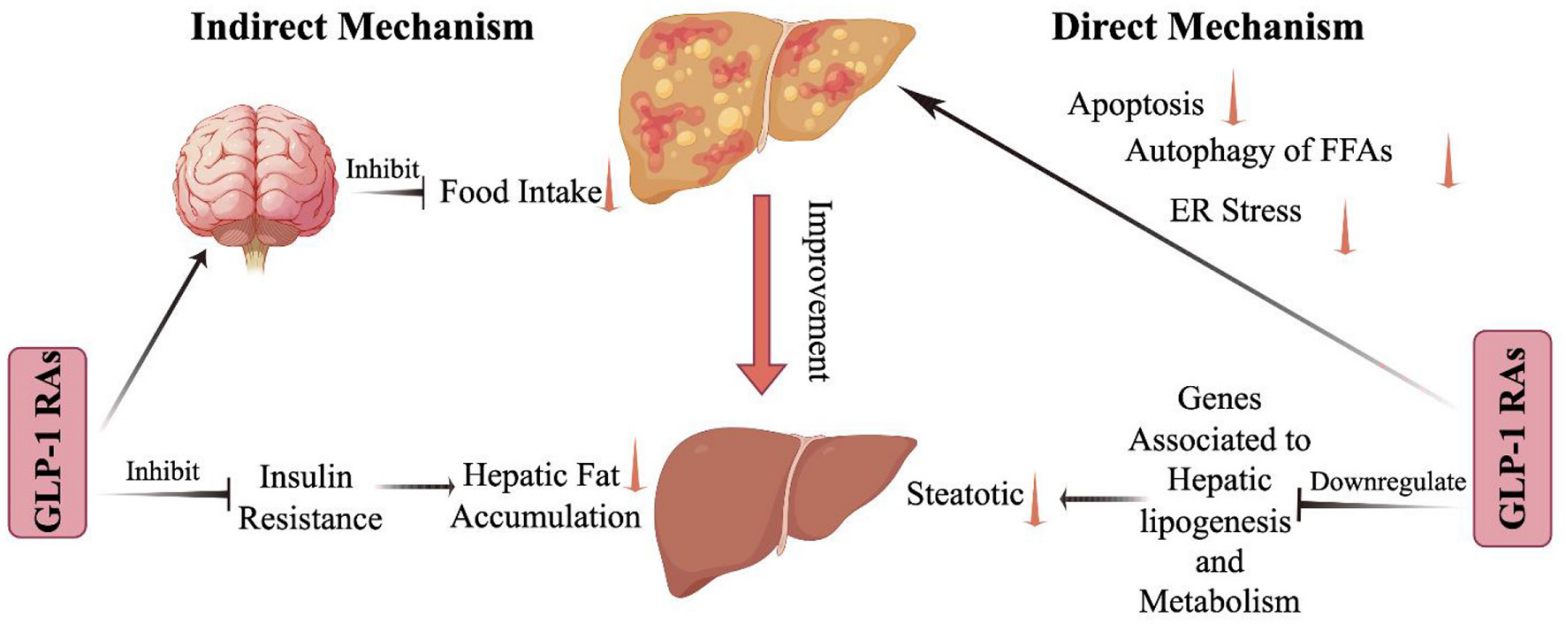
The underlying mechanism of action of GLP-1 RAs in MASLD.

GLP-1 is an intestinal peptide hormone primarily produced and secreted by intestinal enteroendocrine L-cells (35). GLP-1R is present in the human pancreas, several brain regions responsible for appetite, satiety, and food intake-energy balance, and the gastrointestinal tract (36–38). The pathophysiological mechanisms by which GLP-1 RAs improve MASLD can be classified into two categories, the underlying mechanism of action of GLP-1 RAs in MASLD was shown in Figure 6. First, the effects of GLP-1 RAs in the liver are mediated by indirect pleiotropic mechanisms (39, 40); Second, ligand-receptor interactions may play a role; however, this is still being debated (37).

GLP-1 RAs systematically improve metabolic processes by activating GLP-1 Rs expressed in multiple organs, thereby indirectly improving hepatic pathological changes during MASLD. GLP-1 RAs reduce food intake and inhibit gastric emptying by activating GLP-1R+ cells in the nervous system, resulting in weight loss (41). Simultaneously, weight loss and improvement in fibrosis were observed in MASLD (42). GLP-1 RAs also inhibit fat accumulation by reducing the shuttling of free fatty acids to the liver. White adipose tissue is primarily used to store excess fat within the body and is widely distributed in the subcutaneous tissue and around the internal organs. Experimental studies on MASLD/MASH mice have demonstrated that, as insulin resistance develops in MASLD, postprandial lipolysis of white adipocytes increases, releasing fatty acids into the bloodstream and leading to an increase in *de novo* lipogenesis (DNL) in the liver (43). Insulin resistance in the adipose tissue was improved by GLP-1 RAs treatment, which further suppressed lipolysis and decreased hepatic DNL *in vivo* (44–46). Semaglutide improved histological markers of fibrosis and inflammation and reduced the hepatic expression of fibrosis- and inflammation-related gene pathways in two preclinical MASH models (47). Seventy-two proteins, most of which were related to metabolism, fibrosis, and inflammation, were significantly associated with MASH resolution and semaglutide treatment. These findings suggest that semaglutide protects the liver, independent of weight loss.

In contrast, GLP-1R is present on human hepatocytes, which may regulate hepatic pathological processes directly in MASLD. GLP-1RA reduces steatosis and improves mitochondrial function and survival of primary human hepatocytes *in vitro* by reducing apoptosis and fatty acid-induced endoplasmic reticulum (ER) stress, and inducing autophagy of free fatty acids (FFAs) in treated cells (10). Furthermore, GLP-1 RAs may modulate hepatoprotective and antisteatotic effects by downregulating genes associated with key pathways of hepatic lipogenesis and fatty acid metabolism, such as *PPAR*γ, *ACSL1, ApoB*, and *SHP1* (48, 49). Additionally, *in vivo* and *in vitro* studies have shown that liraglutide treatment can ameliorate hepatic lipid accumulation by activating AMPK (49, 50). However, conflicting results have also been reported; therefore, exploring the molecular mechanism of GLP-1 RAs in MASLD is essential to provide more accurate guidance for clinical applications.

### 4.3 Limitation

To the best of our knowledge, only a few umbrella reviews have evaluated the efficacy and safety of GLP-1 RAs in MASLD. This review provides a multidimensional summary of the efficacy of GLP-1 RAs in MASLD, examining key dimensions to create a comprehensive evidence map of treatment with GLP-1 RAs in MASLD. The efficacy of different types of GLP-1 RAs is also discussed, providing a crucial evidence-based decision-making basis for formulating clinical guidelines and optimizing treatment strategies.

However, this study has several limitations. First, the sample size of most of the included studies was < 1,000, resulting in insufficient overall statistical power to detect potentially significant effects and highlighting the need for conducting large-scale multi-center randomized controlled trials. Additionally, nearly 80% of the included reviews were rated as critically low quality by AMSTAR 2. This fundamentally undermines the reliability of our conclusions and underscores the urgent need for high-quality primary studies. Furthermore, most reviews lacked subgroup analyses for the different types of GLP-1 RAs, the presence of T2DM, and dose. Consequently, the reported summary effect for an intervention may represent an average, which may not accurately reflect its efficacy in either subgroup. Future studies should conduct patient-data-based or refined network meta-analyses of different GLP-1 RAs, doses, and MASLD populations with or without T2DM to distinguish their efficacy and safety. Finally, a moderate-to-high overlap (CCA = 10.47%) was observed, meaning limited original studies are repeatedly analyzed across reviews, potentially overestimating the evidence. Future umbrella reviews should apply stricter screening or re-analyze based on primary studies.

## 5 Conclusion

In conclusion, this review suggests that GLP-1 RAs improve MASLD in five dimensions. All types of GLP-1 RAs also showed therapeutic potential for MASLD, and the efficacy of semaglutide in MASLD was highlighted. Additionally, GLP-1 RAs are generally well-tolerated in MASLD. However, nearly 80% of included reviews were of critically low quality (AMSTAR 2) and there was moderate-to-high evidence overlap (CCA 10.47%), which fundamentally limits the confidence in these findings. Due to the lack of sub-analyses by dose or T2DM status, only an average summary effect could be reported. Therefore, our conclusions should be viewed as preliminary and hypothesis-generating. High-quality primary studies are urgently needed to investigate the optimal types/doses of GLP-1 RAs and their underlying mechanisms in MASLD.

## Data Availability

The original contributions presented in the study are included in the article/Supplementary material, further inquiries can be directed to the corresponding authors.
