# Supplementary material for: Efficacy and safety of GLP-1 receptor agonists in patients with metabolic-associated fatty liver diseases: an umbrella review
Source: Front Med (Lausanne). 2026 May 28;13:1783429. doi: 10.3389/fmed.2026.1783429 (PMC13253451; doi:10.3389/fmed.2026.1783429)
Supplement: Supplementary file 1 [file Supplementary_File_1.docx]

**Supplementary Material 1**

**Supplementary Table S1.** Search strategy until 30 September 2025.

| **Supplementary Table S2.** Studies excluded after full-text assessment. |
| --- |
| **Patients not diagnosed with MAFLD (n=4)** |
| 1. Rivera FB, Arias-Aguirre E, Aguirre Z, et al. Evaluating the safety profile of semaglutide: an updated meta-analysis. Article. Current Medical Research and Opinion. 2024;40(9):1495-1514. doi:10.1080/03007995.2024.2383731 2. Simental-Mendía LE, Simental-Mendía M, Barragán-Zúñiga LJ, Arce-Quiñones M. Effect of saroglitazar on glycaemic parameters: A systematic review and meta-analysis of randomized controlled trials. Review. Diabetes, Obesity and Metabolism. 2025;27(9):4627-4642. doi:10.1111/dom.16506 3. 1.Liao C, Liang X, Zhang X, Li Y. 1.The effects of GLP-1 receptor agonists on visceral fat and liver ectopic fat in an adult population with or without diabetes and nonalcoholic fatty liver disease: A systematic review and meta-analysis. Article. PLOS ONE. 2023;18(8 August)e0289616. doi:10.1371/journal.pone.0289616 4. 2. Mahdi M, Mahdi A, Al Souky N, et al. A SYSTEMATIC REVIEW ON THE IMPACT OF GLP-1 AGONISTS ON NAFLD: A PROMISING NNT THAT CALLS FOR EARLY SCREENING AND TREATMENT. Gastroenterology. May 2024;166(5):S1643-S1643. |
| **Intervention not about GLP-1 Receptor Agonists (n=23)** |
| 1. Albert SG, Wood EM. Meta-analysis of trials in non-alcoholic fatty liver disease with therapeutic interventions for metabolic syndrome. Article. Diabetes and Metabolic Syndrome: Clinical Research and Reviews. 2021;15(5)102232. doi:10.1016/j.dsx.2021.102232 2. Ampuero J, Gallego-Durán R, Maya-Miles D, et al. Systematic review and meta-analysis: analysis of variables influencing the interpretation of clinical trial results in NAFLD. Article. Journal of Gastroenterology. 2022;57(5):357-371. doi:10.1007/s00535-022-01860-0 3. Armes S, Tibaes J, Rajaram R, et al. Prevalence of polypharmacy and associated side effects in individuals with metabolic dysfunction-associated steatotic liver disease (MASLD): a systematic review and meta-analysis. Article. BMJ Nutrition, Prevention and Health. 2025;doi:10.1136/bmjnph-2025-001236 4. Fu CE, Ng CH, Yong JN, et al. A Meta-analysis on Associated Risk of Mortality in Nonalcoholic Fatty Liver Disease. Article. Endocrine Practice. 2023;29(1):33-39. doi:10.1016/j.eprac.2022.10.007 5. Musso G, Cassader M, Rosina F, Gambino R. Impact of current treatments on liver disease, glucose metabolism and cardiovascular risk in non-alcoholic fatty liver disease (NAFLD): A systematic review and meta-analysis of randomised trials. Review. Diabetologia. 2012;55(4):885-904. doi:10.1007/s00125-011-2446-4 6. Xie Q, Pan X, Zhang X, Ma J, Peng G, Tong N. Histological analysis of hypoglycemic agents on liver fibrosis in patients with non-Alcoholic fatty liver disease: A systematic review. Letter. Chinese Medical Journal. 2023;136(16):2014-2016. doi:10.1097/CM9.0000000000002778. 7. Biciusca T, Stan SI, Balteanu MA, et al. The Role of the Fatty Liver Index (FLI) in the Management of Non-Alcoholic Fatty Liver Disease: A Systematic Review. Review. Diagnostics. 2023;13(21)doi:10.3390/diagnostics13213316 8. Blazina I, Selph S. 8.Diabetes drugs for nonalcoholic fatty liver disease: A systematic review. Article. Systematic Reviews. 2019;8(1)doi:10.1186/s13643-019-1200-8 9. Carbone LJ, Angus PW, Yeomans ND. Incretin-based therapies for the treatment of non-alcoholic fatty liver disease: A systematic review and meta-analysis. Article. Journal of Gastroenterology and Hepatology (Australia). 2016;31(1):23-31. doi:10.1111/jgh.13026 10. Dar S, Siddiqi AK, Alabduladhem TO, et al. Effects of novel glucose-lowering drugs on the lipid parameters: A systematic review and meta-analysis. Review. Annals of Medicine and Surgery. 2022;77103633. doi:10.1016/j.amsu.2022.103633 11. Dougherty JA, Guirguis E, Thornby KA. 4.A Systematic Review of Newer Antidiabetic Agents in the Treatment of Nonalcoholic Fatty Liver Disease. Review. Annals of Pharmacotherapy. 2021;55(1):65-79. doi:10.1177/1060028020935105 12. Dutta D, Surana V, Singla R, Aggarwal S, Sharma M. Efficacy and safety of novel twincretin tirzepatide a dual GIP and GLP-1 receptor agonist in the management of type-2 diabetes: A Cochrane meta-analysis. Review. Indian Journal of Endocrinology and Metabolism. 2021;25(6):475-483. doi:10.4103/ijem.ijem_423_21 13. Kamrul-Hasan ABM, Dutta D, Nagendra L, Kuchay MS, Islam MS, Pappachan JM. Hepatobiliary effects and safety of tirzepatide: A systematic review and meta-analysis. Letter. Diabetes, Obesity and Metabolism. 2024;26(12):6074-6079. doi:10.1111/dom.15948 14. Khanmohammadi S, Habibzadeh A, Kamrul-Hasan ABM, Schuermans A, Kuchay MS. Glucose-lowering drugs and liver-related outcomes among individuals with type 2 diabetes: A systematic review of longitudinal population-based studies. Review. Diabetic Medicine. 2024;41(11)e15437. doi:10.1111/dme.15437 15. Konings LAM, Miguelañez-Matute L, Boeren AMP, et al. 6.Pharmacological treatment options for metabolic dysfunction-associated steatotic liver disease in patients with type 2 diabetes mellitus: A systematic review. Review. European Journal of Clinical Investigation. 2025;55(4)e70003. doi:10.1111/eci.70003 16. Kumar J, Memon RS, Shahid I, et al. 5.Antidiabetic drugs and non-alcoholic fatty liver disease: A systematic review, meta-analysis and evidence map. Review. Digestive and Liver Disease. 2021;53(1):44-51. doi:10.1016/j.dld.2020.08.021 17. Leoni S, Tovoli F, Napoli L, Serio I, Ferri S, Bolondi L. Current guidelines for the management of non-alcoholic fatty liver disease: A systematic review with comparative analysis. Review. World Journal of Gastroenterology. 2018;24(30):3361-3373. doi:10.3748/wjg.v24.i30.3361 18. Lucas C, Lucas G, Lucas N, Krzowska-Firych J, Tomasiewicz K. A systematic review of the present and future of non-alcoholic fatty liver disease. Review. Clinical and Experimental Hepatology. 2018;4(3):165-174. doi:10.5114/ceh.2018.78120 19. Maiorino MI, Chiodini P, Bellastella G, Capuano A, Esposito K, Giugliano D. Insulin and glucagon-like peptide 1 receptor agonist combination therapy in type 2 diabetes: A systematic review and meta-analysis of randomized controlled trials. Review. Diabetes Care. 2017;40(4):614-624. doi:10.2337/dc16-1957 20. Mantovani A, Byrne CD, Targher G. Efficacy of peroxisome proliferator-activated receptor agonists, glucagon-like peptide-1 receptor agonists, or sodium-glucose cotransporter-2 inhibitors for treatment of non-alcoholic fatty liver disease: a systematic review. Review. The Lancet Gastroenterology and Hepatology. 2022;7(4):367-378. doi:10.1016/S2468-1253(21)00261-2 21. Qin W, Yang J, Ni Y, et al. Efficacy and safety of once-weekly tirzepatide for weight management compared to placebo: An updated systematic review and meta-analysis including the latest SURMOUNT-2 trial. Article. Endocrine. 2024;86(1):70-84. doi:10.1007/s12020-024-03896-z 22. Wang Y, Zhou Y, Wang Z, Ni Y, Prud'homme GJ, Wang Q. X55.Efficacy of GLP-1-based Therapies on Metabolic Dysfunction-associated Steatotic Liver Disease and Metabolic Dysfunction-associated Steatohepatitis: A Systematic Review and Meta-analysis. J Clin Endocrinol Metab. Sep 16 2025;110(10):2964-2979. doi:10.1210/clinem/dgaf336 23. Wong C, Yaow CYL, Lee MH, et al. A META-ANALYSIS OF SODIUM-GLUCOSE CO-TRANSPORTER-2 INHIBITORS AND GLUCAGON-LIKE PEPTIDE-1 RECEPTOR AGONISTS FOR NON-ALCOHOLIC FATTY LIVER DISEASE IN PATIENTS WITH TYPE 2 DIABETES MELLITUS. Gastroenterology. May 2021;160(6):S839-S840. |

**Supplementary Fig S1.** The publish time distribution of included meta-analysis and systematic reviews.


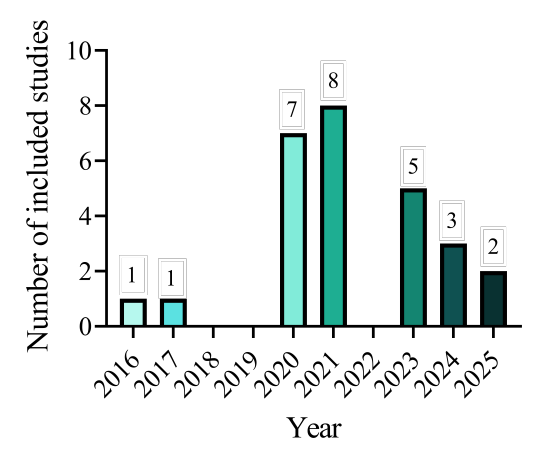


**Supplementary Fig S2.** The number of included meta-analysis and systematic reviews classified by different kinds of GLP-1 RAs.


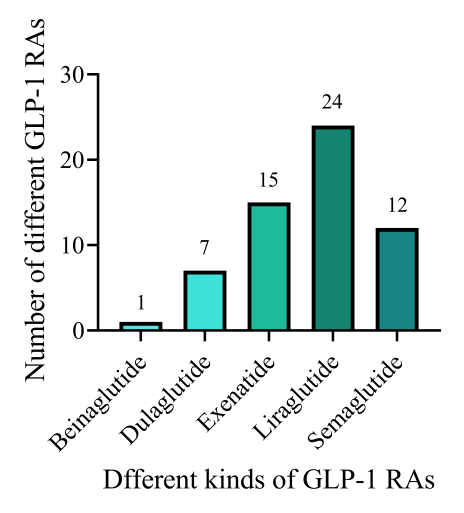


**Supplementary Table S3.** Results of Overlap analysis of included systematic reviews.


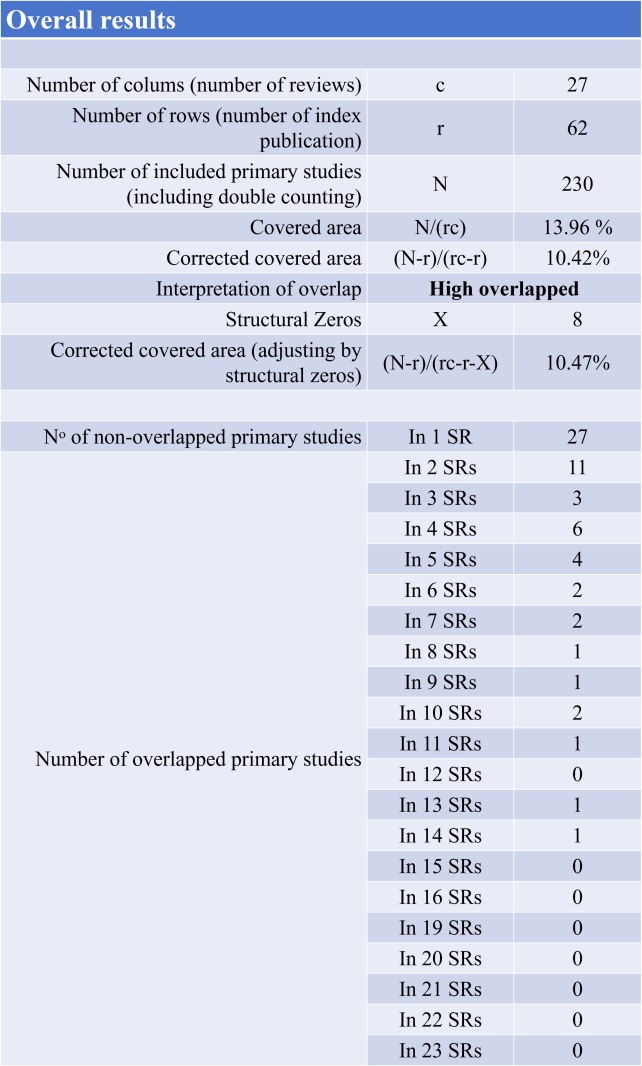


| **Supplementary Table S4.** Characteristics of primary studies. | | | | | | | | | | |  |
| --- | --- | --- | --- | --- | --- | --- | --- | --- | --- | --- | --- |
|  | **NO.** | **Author** | **Year** | **Country** | **Sample size (n)** | **Dose^a^** | **Histological resolution rate of GLP-1 RAs** | **The change in HFC** | **Main AE and Its Incidence rate of GLP-1 RAs** | |  |
| **Liraglutide** | 1 | Armstrong et al. | 2016 | UK | 52 (26 for liraglutide, 26 for placebo) | 1.8 mg qd w1~48 | 39% (9/22) | NA | Diarrhoea  (38%, 10/22) | |  |
|  | 2 | FENG et al. | 2017 | China | 93 (30 for liraglutide, 32 for gliclazide, and 31 for metformin) | 0.6 mg qd w1; 1.2 mg qd w2; 1.8 mg qd w3~24 | NA | From 36.70±3.65% to 13.11±1.84% (P=0.001) | Appetite suppression (73.3%, 22/30) | |  |
|  | 3 | MBBS et al. | 2017 | Singapore | 24 (12 for liraglutide, 12 for DE) | 0.6 mg qd and increased by 0.6 mg weekly until 3 mg; 3 mg qd w5~22 | NA | -7.2%±7.1% | Abdominal discomfort  and bloating | |  |
|  | 4 | Khoo et al. | 2019 | Singapore | 30 (15 for liraglutide, 15 for DE) | 0.6 mg qd and increased by 0.6 mg weekly until 3 mg; 3 mg qd w5~22 | NA | -7.0±7.1% | Abdominal discomfort  and bloating (100%, 15/15) | |  |
|  | 5 | Yan et al. | 2018 | China | 75 (30 for metformin add-on liraglutide, 32 for add-on sitagliptin, and 31 for add-on insulin) | 1.8 mg qd w1~26 | NA | -15.4%±5.6% | NA | |  |
|  | 6 | Zhang et al. | 2020 | China | 60 (30 for liraglutide, 30 for pioglitazone) | 0.6 mg qd w1; 1.2 mg qd w2~24 | NA | From 24.1±3.0 to 20.1± 3.8 (P < 0.05) | Gastrointestinal reactions (30%, 9/30 ) | |  |
|  | 7 | Tian et al. | 2018 | China | 127 (52 for liraglutide, 75 for metformin) | 0.6~1.2 mg qd w1~12 | NA | NA | Moderate gastrointestinal disturbances (17.3%, 9/52) | |  |
|  | 8 | Feng et al. | 2019 | China | 85 (29 for liraglutide, 27 for gliclazide, and 29 for metformin) | 0.6 mg qd w1; 1.2 mg qd w2; 1.8 mg qd w3~24 | NA | NA | Diarrhea (13.8%, 4/29) | |  |
|  | 9 | Eguchi et | 2015 | Japan | 27 (26 completed the | 0.9 mg qd | 66.67%(6/9) | NA | NA | |  |
| **Supplementary Table S4 (continued)** | | | | | | | | | | |  |
| **Liraglutide** |  | al. |  |  | DE intervention, 19 received liraglutide) | w25~48 |  |  |  | |  |
|  | 10 | Armstrong et al. | 2015 | UK | 14 (7 for liraglutide, 7 for placebo) | 0.6 mg qd w1; 1.2 mg qd w2; 1.8 mg qd w3~12 | NA | NA | NA | |  |
|  | 11 | Smits et al. | 2016 | Netherlands | 52 (17 for liraglutide, 18 for sitagliptin, and 17 for placebo) | 1.8 mg qd w1~12 | NA | -10% | | NA | |
|  | 12 | Bizino et al. | 2020 | Netherlands | 50 (24 for liraglutide, 26 for placebo) | 0.6 mg qd w1; 1.2 mg qd w2; 1.8 mg qd w3~26 | NA | NA | | NA | |
|  | 13 | Guo et al. | 2020 | China | 96 (32 for liraglutide, 32 for insulin, and 32 for placebo) | 0.6 mg qd w1; 1.2 mg qd w2; 1.8 mg qd w3~26 | NA | From 26.4%±3.2% to 20.6%± 3.9% (P < 0.05) | | Nausea and vomiting (25.8%, 8/32) | |
|  | 14 | Matikainen et al. | 2018 | Finland | 22 (15 for liraglutide, 7 for placebo) | 1.8 mg qd w1~16 | NA | -31% | NA | |  |
|  | 15 | Bouchi et al. | 2016 | Japan | 19 (9 for liraglutide with insulin, 10 for insulin) | 0.3 mg qd w1; 0.6 mg qd w2; 0.9 mg qd w3~24 | NA | NA | NA | |  |
|  | 16 | Tang et al. | 2015 | Canada | 35 (18 for liraglutide, 17 for insulin) | 0.6 mg qd w1; 1.2 mg qd w2; 1.8 mg qd w3~12 | NA | From 13.8% to 10.6% (P = 0.005) | Nausea and diarrhea (5.6%, 1/18); Nausea and  Vomiting (5.6%, 1/18,); Headaches (5.6%, 1/18) | |  |
|  | 17 | Vanderheiden et al. | 2016 | USA | 71 (35 for liraglutide, 36 for placebo) | 0.6 mg qd w1; 1.2 mg qd w2; 1.8 mg qd w3~24 | NA | A significant decrease in the liraglutide group compared with the placebo group (P = 0.0006) | NA | |  |
|  | 18 | Ohki et al. | 2012 | Japan | 82 (26 for liraglutide, 36 for sitagliptin, and 20 for pioglitazone) | 0.3 mg qd w1; 0.6 mg qd w2; 0.9 mg qd w3~ | NA | NA | NA | |  |
|  | 19 | Vedtofte et al. | 2020 | Denmark | 82 (37 for liraglutide, 45 for placebo) | 0.6 mg qd w1; 1.2 mg qd w2; 1.8 mg | NA | NA | NA | |  |
| **Supplementary Table S4 (continued)** | | | | | | | | | | |  |
| **Liraglutide** |  |  |  |  |  | qd w3~48 |  |  |  | |  |
|  | 20 | Petit et al. | 2016 | France | 57 | 0.6 mg qd w1; 1.2 mg qd w2~24 | NA | -33% (From 20.1% to 13.5%;  P<0.0001) | NA | |  |
|  | 21 | Frøssing et al. | 2017 | Denmark | 72 (48 for liraglutide, 24 for placebo) | 1.8 mg qd w1~26 | NA | -44% | Nausea (79%); Constipation (26%) | |  |
|  | 22 | Zhang et al. | 2018 | China | 835 (424 for liraglutide, 411 for conventional drug) | 1.2 mg qd w1~12 | NA | NA | NA | |  |
|  | 23 | Smits et al. | 2017 | Netherlands | 55 (19 for liraglutide, 19 for sitagliptin and 17 for placebo) | Not about MAFLD | | | | |  |
|  | 24 | Jendle et al. | 2009 | UK | 160 | Not about MAFLD | | | | |  |
| **Beinaglutide** | 25 | Fan et al. | 2023 | China | 50 (25 for beinaglutide, 25 for lifestyle intervention) | 0.1 mg tid w1~24 | NA | IHTG content ≥ 50% in the beinaglutide group was  4.35 times than that in the lifestyle intervention group (OR 4.35, 95%CI[1.13, 16.85]; P = 0.028) | NA | |  |
| **Dulaglutide** | 26 | Seko et al. | 2017 | Japan | 15 | 0.75 mg w1~12 | NA | NA | Diarrhea (6.7%, 1/15,) | |  |
|  | 27 | Kuchay et al. | 2020 | India | 64 (34 for dulaglutide add-on to usual care, 34 for usual care) | 0.75 mg qw w1~4; 1.5 mg qw w5~20 | NA | −32.1%  (control: −5.7%; MD=−26.4%, 95%CI[−44.2, −8.6%], P = 0.004) | Gastrointestinal upset (17.7%, 6/34 ) | |  |
| **Exenatide** | 28 | Liu et al. | 2020 | China | 76 (38 for exenatide, 38 for insulin glargine) | 5 ug bid w1~4; 10 ug bid w5~24 | NA | −17.55±12.93% (control: −10.49±11.38%, P=0.1248) | No significant difference between two group | |  |
|  | 29 | Shao et al. | 2014 | China | 60 (30 for exenatide with insulin glargine, | 5 ug bid w1~4; 10 ug bid w5~12 | NA | The reversal rate of fatty liver 93.3% (control: 66.7%, | NA | |  |
| **Supplementary Table S4 (continued)** | | | | | | | | | | |  |
|  | 29 |  |  |  | 30 for insulin aspart with insulin glargine) |  |  | P<0.01) |  | |  |
| **Exenatide** | 30 | Ruff et al. | 2022 | USA | 4156 (2075 for exenatide, 2081 for placebo) | Not about MAFLD | | | | |  |
|  | 31 | Fan et al. | 2013 | China | 117 (49 for exenatide, 68 for metformin) | 5 ug bid w1~4; 10 ug bid w5~12 | NA | NA | NA | |  |
|  | 32 | Bi et al. | 2014 | China | 33 (for exenatide, insulin and pioglitazon) | 5 ug bid w1~4; 10 ug bid w5~24 | NA | −68% (insulin: -58%, pioglitazon: -49%) | NA | |  |
|  | 33 | Savvidou et al. | 2015 | Greece | 127 | 5 ug bid w1~4; 10 ug bid w5~24 | NA | NA | NA | |  |
|  | 34 | Kenny et al. | 2010 | USA | 8 | 5 ug bid w1~3; 10 ug bid w4~28 | 50% (4/8) | NA | Nausea and abdominal pain (25%, 2/8) | |  |
|  | 35 | Sathyanarayana et al. | 2011 | USA | 21 (11 for exenatide with pioglitazon, 10 for pioglitazon) | 5 ug bid w1~2; 10 ug bid w3~53 | NA | From 12.1±1.7% to 4.7 ± 1.3% (control: From 11.0±3.1% to 6.5±1.9%) | NA | |  |
|  | 36 | Dutour et al. | 2016 | France | 44 (for exenatide or reference treatment) | 5 ug bid w1~4; 10 ug bid w5~26 | NA | -23.8±9.5% (control: -12.5±9.6%, p=0.007) | NA | |  |
|  | 37 | Blaslov et al. | 2014 | NA | 125 (87 for exenatide ) | NA | NA | −25.95 ± 23.15 | NA | |  |
|  | 38 | Buse et al. | 2007 | USA | 38 | 5 ug bid | NA | NA | NA | |  |
| **Semaglutide** | 39 | Newsome et al. | 2021 | UK | 320 (subcutaneous semaglutide at a dose of 0.1, 0.2, or 0.4 mg or corresponding placebo in a 3:3:3:1:1:1 ratio) | 0.1, 0.2, or 0.4 mg qd w1~72 | 59% in the 0.4mg group (17% in the placebo group; OR 6.87; 95%CI[2.60, 17.63]; P<0.001) | NA | Nausea, 42%; Constipation, 22%; Vomiting, 15%; Malignant neoplasms, 1% (the placebo group: 11%; 12%; 2%; 0%) | |  |
|  | 40 | Flint et al. | 2021 | Greece | 67 (34 for semaglutide, 33 for placebo) | Initial 0.05 mg qd w1~4; Increase every 4 weeks (0.1, 0.2 and 0.3 | NA | NA | NA | |  |
| **Supplementary Table S4 (continued)** | | | | | | | | | | |  |
| **Semaglutide** |  |  |  |  |  | mg/day) to reach a target dose of 0.4 mg qd by week 16 |  |  |  | |  |
|  | 41 | Loomba et al. | 2023 | USA | 71 (47 for semaglutide, 24 for placebo) | Initial 0.24 mg to 0.5 mg w1~4, and thereafter every  4 weeks to 1.0 mg, 1.7 mg, and finally 2.4 mg once weekly after 16 weeks’ treatment | 11% (5/47; placebo: 29%, 7/24) | NA | Nausea (45%, 21/47) | |  |
|  | 42 | Alkhouri et al. | 2022 | USA | 108 (21 for semaglutide, 22 for semaglutide plus cilofexor, 222for semaglutide plus cilofexo, 22 for semaglutide plus firsocostat, 21 for semaglutide, cilofexor and firsocostat) | Initial 0.24 mg, thereafter every  4 weeks to 0.5 mg, 1.0 mg and 1.7 mg until 2.4 mg was reached (from week 17 onwards) | NA | NA | Nausea (43%) | |  |
|  | 43 | Newsome et al. | 2018 | UK | 957 | 0.05, 0.1,  0.2, 0.3 or 0.4 mg qd w1~w52 (initial with 0.05 mg qd, sequentially escalate every 4 weeks) | NA | NA | NA | |  |
|  | 44 | Gómez et | 2023 | USA | 145 (72 for | 0.25 mg qw | NA | -42.3% (efinopegdutide: | Nausea (31.5%, 23/73) | |  |
| **Supplementary Table S4 (continued)** | | | | | | | | | | |  |
| **Semaglutide** |  | al. |  |  | efinopegdutide, 73 for semaglutide) | w1~4; 0.5 mg qw w5~8; 1 mg qw w9~24 |  | 72.7%) |  | |  |
|  | 45 | Neil et al. | 2018 | UK | 975 | Not about MAFLD | | | | |  |
|  | 46 | Arai et al. | 2022 | Japan | 16 | 3mg, qw, w1~3; 7mg, qw, w4~8; 14mg, qw, w9~24 (oral) | NA | NA | Nausea (50%, 8/16) | |  |
|  | 47 | Gómez et al. | 2022 | Spain | 213 | 0.5mg, qw, w1~24; 1mg, qw, w1~24 | NA | NA | Nausea (22.5%, 48/213); Diarrhea (7.5%, 16/213); Hypoglycemic episodes (9.9%, 21/213) | |  |
|  | 48 | Volpe et al. | 2022 | Italy | 48 | 0.25mg, qw, w1~4; 0.5mg, qw, w5~24; 1mg, qw, w6~ | NA | NA | NA | |  |
| **Tirzepatide** | 49 | Gastaldelli et al. | 2022 | Spain | 502 | Not about GLP-1 RAs- | | | | |  |
| **Liraglutide & Exenatide** | 50 | Cuthbertson et al. | 2012 | UK | 25 (6 for liraglutide, 19 for exenatide) | Liraglutide: 1~2mg, qd, w1~24; Exenatide: 5ug, bid, w1~4; 10ug, bid, w5~24 | NA | -42% | NA | |  |
|  | 51 | Díaz et al. | 2015 | Spain | 58 (15 for gliclazide, 13 for pioglitazone, 15 for sitagliptin, 7 for exenatide, 8 liraglutide | Liraglutide: 0.6mg, qd, w1; 1.2mg, qd, w2~24  Exenatide: 5ug, bid, w1~4; 10ug, bid, w5~24 | NA | NA | NA | |  |
| **Liraglutide & Exenatide &** | 52 | Makri et al. | 2020 | Greece | 189 (29 for exenatide, 6 for dulaglutide, 2 | Liraglutide: 1.2mg, qd; | NA | NA | NA | |  |
| **Supplementary Table S4 (continued)** | | | | | | | | | | |  |
| **Dulaglutide** |  |  |  |  | for liraglutide) | Dulaglutide:1.5mg, qw;  Exenatide: 1ug, bid, w5~24 |  |  |  | |  |
|  | 53 | Lee et al. | 2012 | Belonging to secondary analysis | | | | | | |  |
|  | 54 | Balena et al. | 2013 | Belonging to secondary analysis | | | | | | |  |
|  | 55 | Schuster et al. | 2018 | Belonging to secondary analysis | | | | | | |  |
|  | 56 | Castera et al. | 2019 | Belonging to secondary analysis | | | | | | |  |
|  | 57 | Lefebvre et al. | 2019 | Not about GLP-1 RAs | | | | | | |  |
|  | 58 | Brunt et al. | 2011 | Not about GLP-1 RAs | | | | | | |  |
|  | 59 | Cusi et al. | 2016 | Not about GLP-1 RAs | | | | | | |  |
|  | 60 | Gómez et al. | 2023 | Not about GLP-1 RAs | | | | | | |  |
| Not found | 61 | Not found | | | | | | | | |  |
| Not found | 62 | Not found | | | | | | | | |  |

Histological resolution : the resolution of steatohepatitis (disappearance of hepatocyte ballooning) without worsening of fibrosis (defined as a numerical increase in the stage of the Kleiner fibrosis classification). HFC:Hepatic Fat Content; AE: adverse effects; DE: diet-exercise; IHTG: improving intrahepatic triglyceride; Not Appropriate: NA.

^a^:Unless indicated as oral, the drug was administered by subcutaneous injection.

**Supplementary Figure S3** Regional distribution of primary studies


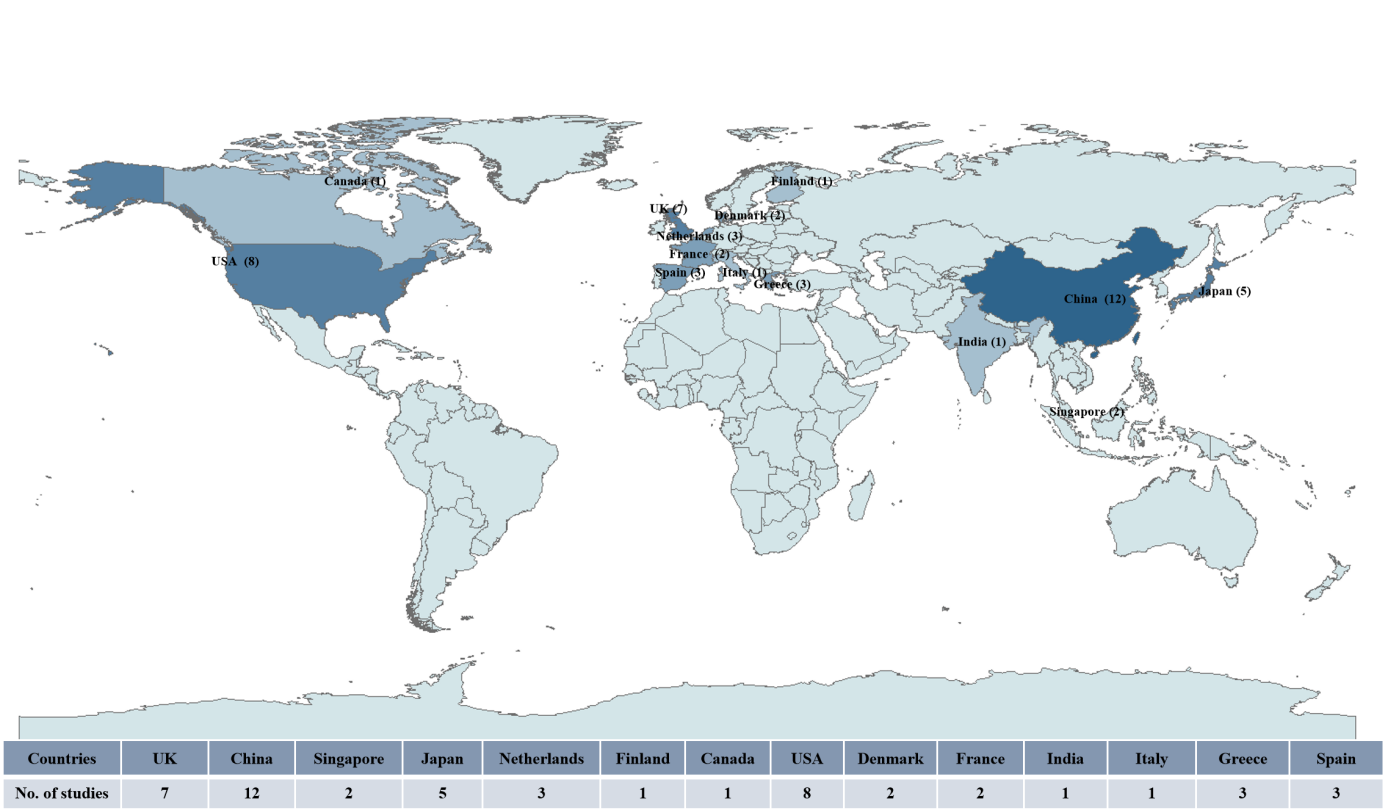


**Figure S4.** Pooled estimation of the effect of GLP-1 RAs on histologic resolution.


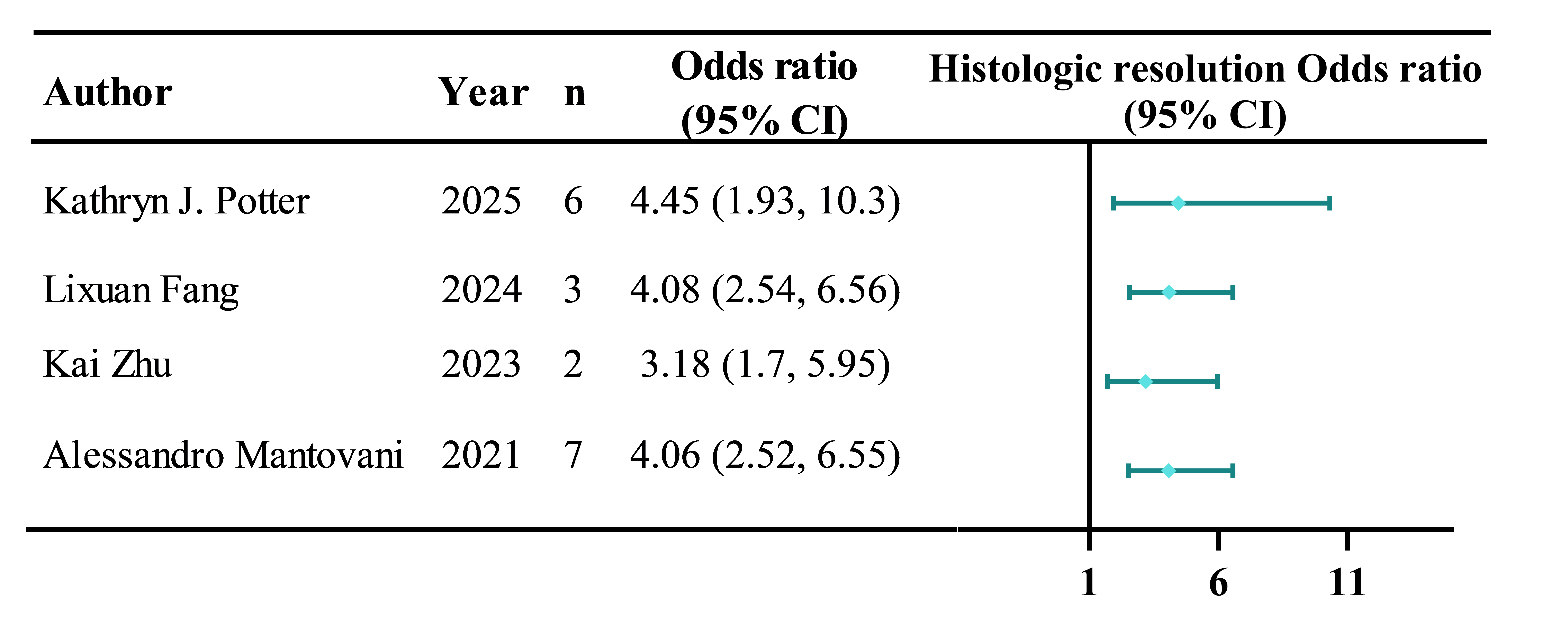


**Supplementary Fig S5.** Pooled estimation of the effect on liver enzyme of liraglutide, semaglutide, exenatide and dulaglutide in MAFLD.



**Supplementary Fig S6.** Pooled estimation of the effect on lipid markers of liraglutide, semaglutide, exenatide and dulaglutide in MAFLD.


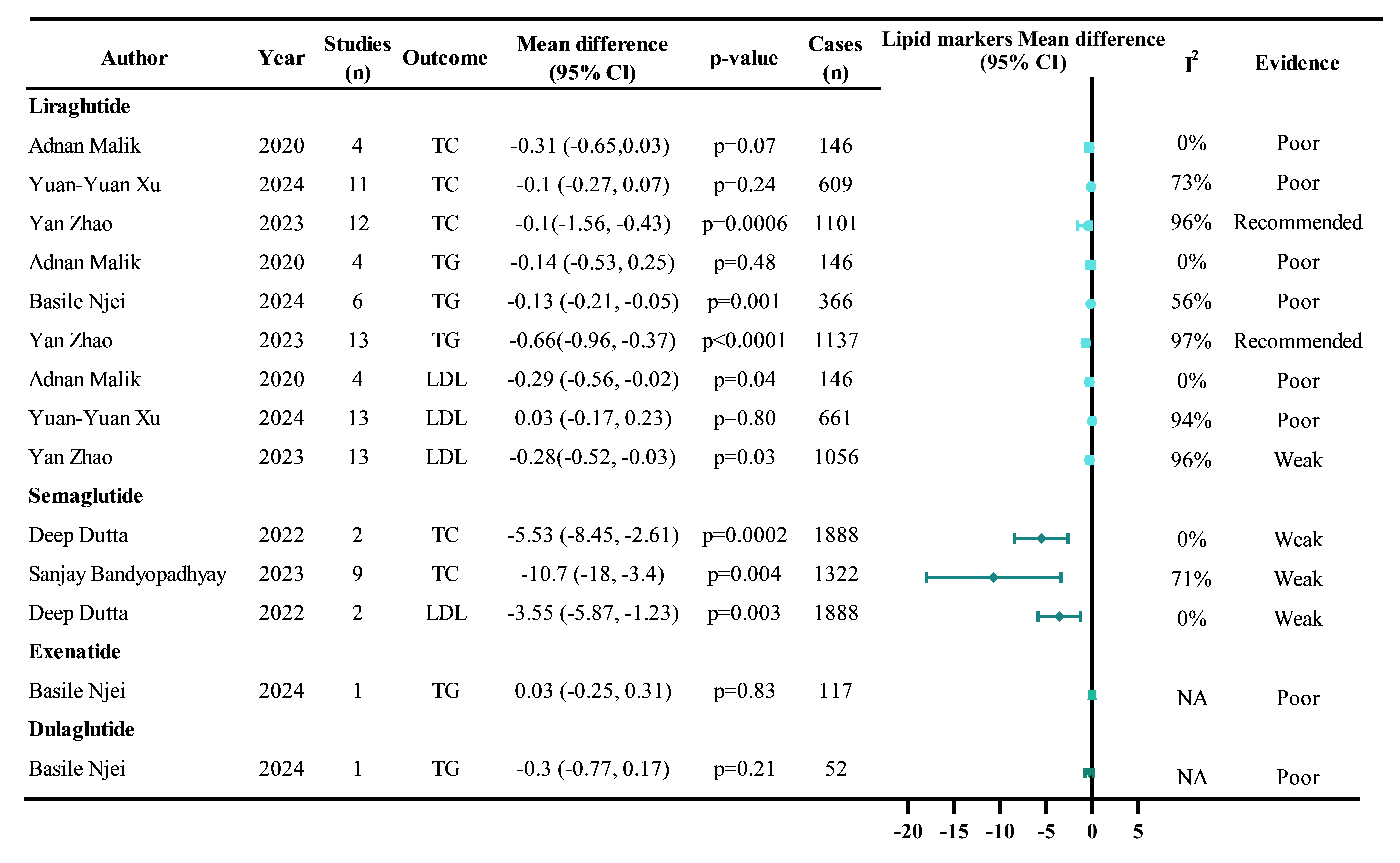


**Supplementary Fig S7.** Pooled estimation of the effect of GLP-1 RAs on HbA1c in MAFLD.


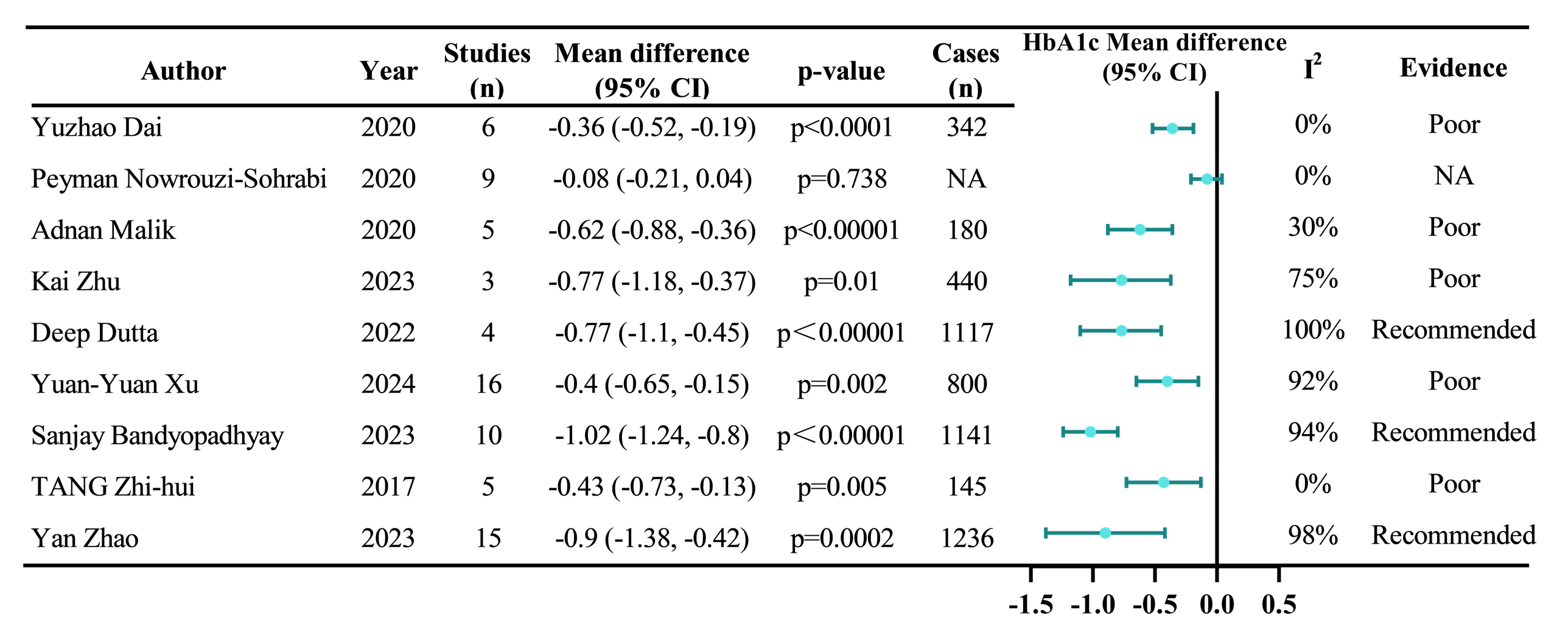


**Supplementary Fig S8.** Pooled estimation of the effect of GLP-1 RAs on anthropometric measuring in MAFLD, including body weight, BMI and WC.





**Supplementary Fig S9.** Pooled estimation of the effect of GLP-1 RAs on fat distribution in MAFLD, including SAT and VAT.


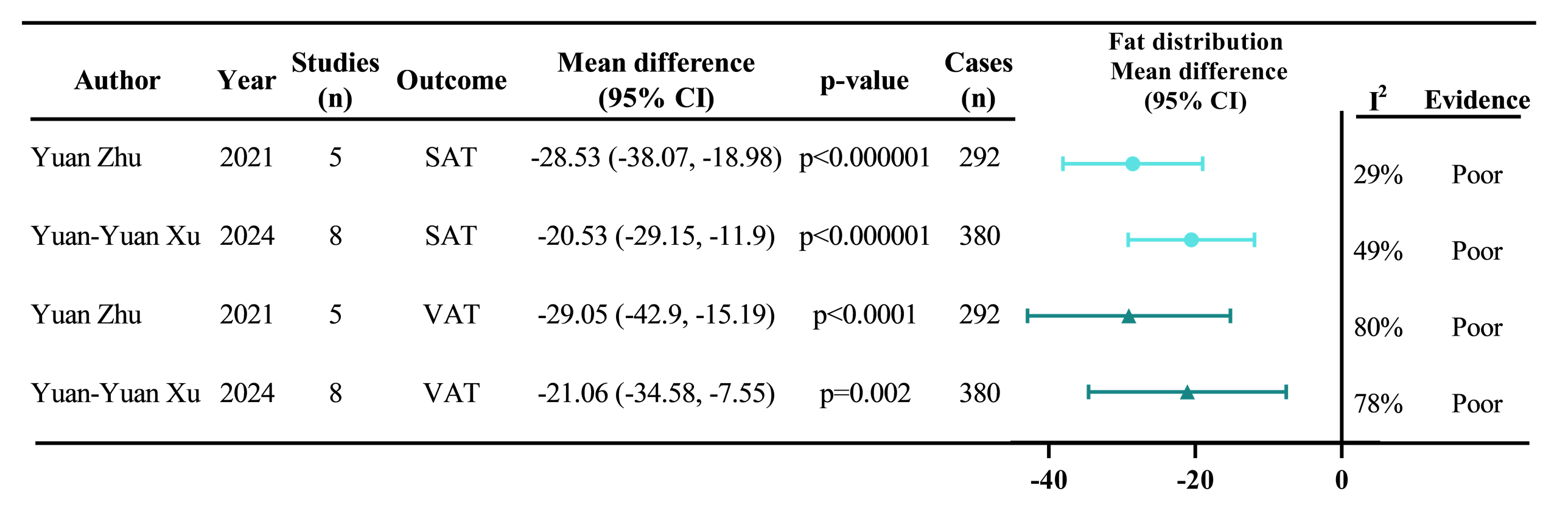


**Supplementary Table S5.** Pooled estimation and epidemiological credibility assessment of the included meta-analyses on the effect of GLP-1 RAs on histologic resolution of MAFLD.

| **No.** | **Author** | **Year** | **Studies (n)** | **Outcome** | **Odds ratio (95% CI)** | **p-value** | **Cases (n)** | **I2** | **Evidence** |
| --- | --- | --- | --- | --- | --- | --- | --- | --- | --- |
| 1 | Kathryn J. Potter | 2025 | 6 | Histologic resolution | 4.45 (1.93, 10.3) | p＜0.0001 | 206 | 84% | Poor |
| 2 | Lixuan Fang | 2024 | 3 | Histologic resolution | 4.08 (2.54, 6.56) | p＜0.00001 | 391 | 0% | Poor |
| 3 | Kai Zhu | 2023 | 2 | Histologic resolution | 3.18 (1.7, 5.95) | p=0.0003 | 301 | 0% | Poor |
| 4 | Alessandro Mantovani | 2021 | 7 | Histologic resolution | 4.06 (2.52, 6.55) | p=0 | 391 | 0% | Poor |

**Supplementary Table S6.** Pooled estimation and epidemiological credibility assessment of the included meta-analyses on the effect of GLP-1 RAs in MAFLD, including HFC, ALT, AST, GGT, ALP, TC, TG, LDL, HbAc1, FBG, HOMA-IR, CRP, Fibrosis-4 index (FIB-4), NAFLD fibrosis score (NHS), body weight, BMI, WC, SAT and VAT.

| **No.** | **Author** | **Year** | **Studies (n)** | **Outcome** | **Mean difference (95% CI)** | **p-value** | **Cases (n)** | **I2** | **Evidence** |
| --- | --- | --- | --- | --- | --- | --- | --- | --- | --- |
| 1 | Yuzhao Dai | 2020 | 6 | HFC | -3.17 (-5.3, -1.03) | p＜0.0001 | 291 | 31% | Poor |
| 2 | Kathryn J. Potter | 2025 | 6 | HFC | -5.09 (-7.49, -2.69) | p＜0.0001 | 206 | 84% | Poor |
| 3 | Tiantian Song | 2021 | 3 | HFC | -1.95 (-4.09, 0.2) | p=0.08 | 127 | 58% | Poor |
| 4 | Basile Nje | 2024 | 24 | HFC | -1.38 (-1.5, -1.26) | p＜0.00001 | 1495 | 80% | Recommended |
| 5 | Yuan Zhu | 2021 | 12 | HFC | -3.01 (-4.75, -1.28) | p=0.0007 | 551 | 65% | Poor |
| 6 | Kai Zhu | 2023 | 2 | HFC | -4.96 (-9.92, 0.01) | p=0.05 | 138 | 64% | Poor |
| 7 | Alessandro Mantovani | 2020 | 4 | HFC | -6.23 (-8.95, -3.51) | p=0 | 230 | 85.90% | Poor |
| 8 | Alessandro Mantovani | 2021 | 7 | HFC | -3.92 (-6.27, -1.56) | p=0 | 371 | 96.66% | Poor |
| 9 | Maria-Styliani Kalogirou | 2020 | 3 | HFC | -3.63 (-9.59, 2.33) | p＜0.00001 | 192 | 96% | Poor |
| 10 | Yuan-Yuan Xu | 2024 | 5 | HFC | -1.18 (-2.85, 0.49) | p=0.0003 | 221 | 81% | Poor |
| 11 | Sanjay Bandyopadhyay | 2023 | 6 | HFC | -4.97 (-6.65, -3.29) | p＜0.00001 | 842 | 90% | Poor |
| 12 | Wu Jianping | 2021 | 4 | HFC | -1.40 (-2.75, -0.05) | P=0.04 | 187 | 0% | Poor |
| 1 | Yuan Zhu | 2021 | 5 | FIB-4 | -0.04 (-0.15, 0.08) | p=0.5 | 238 | 44% | Poor |
| 1 | Yuzhao Dai | 2020 | 7 | ALT | -10.73 (-20.94, -0.52) | p=0.04 | 306 | 74% | Poor |
| 2 | Kathryn J. Potter | 2025 | 4 | ALT | -14.92 (-25.2, -4.65) | p=0.004 | 326 | 84% | Poor |
| 3 | Lixuan Fang | 2024 | 19 | ALT | -11.82 (-16.7, -6.94) | p<0.00001 | 2178 | 93% | Recommended |
| 4 | Adnan Malik | 2020 | 5 | ALT | 2.66 (-1.56, 6.87) | p=0.22 | 180 | 0% | Poor |
| 5 | Tiantian Song | 2021 | 3 | ALT | -1.87 (-4.61, 0.87) | p=0.18 | 83 | 0% | Poor |
| 6 | Basile Njei | 2024 | 25 | ALT | -4.62 (-6.64, -2.6) | p=0.04 | 1424 | 82% | Weak |
| 7 | Yuan Zhu | 2021 | 12 | ALT | -3.82 (-7.04, -0.6) | p=0.02 | 551 | 58% | Poor |
| 8 | Kai Zhu | 2023 | 3 | ALT | -14.06 (-22.06, -6.07) | p=0.0006 | 458 | 0% | Poor |
| 9 | Shahla Rezaei | 2021 | 12 | ALT | -10.14 (-15.84, -4.44) | p=0.000 | NA | 80.60% | Highly recommended |
| 10 | Alessandro Mantovani | 2020 | 7 | ALT | -8.77 (-17.69, 0.14) | p=0.000 | 414 | 87.30% | Poor |
| 11 | Alessandro Mantovani | 2021 | 13 | ALT | -7.21 (-13.35, -1.07) | p=0.02 | 1018 | 78.53% | Weak |
| 12 | Deep Dutta | 2022 | 3 | ALT | -3.89 (-5.41, -2.36) | p<0.00001 | 2050 | 100% | Highly recommended |
| 13 | Maria-Styliani Kalogirou | 2020 | 5 | ALT | -5.66 (-11.53, 0.21) | p=0.06 | 364 | 67% | Poor |
| 14 | Yuan-Yuan Xu | 2024 | 13 | ALT | -3.06 (-6.17, 0.05) | p=0.05 | 690 | 93% | Poor |
| 15 | Sanjay Bandyopadhyay | 2023 | 10 | ALT | -14.07 (-19.39, -8.75) | p<0.00001 | 1336 | 98% | Recommended |
| 16 | TANG Zhi-hui | 2017 | 5 | ALT | -8. 36 (-13. 41, -3. 31) | p=0.001 | 145 | 0% | Poor |
| 17 | Yan Zhao | 2023 | 10 | ALT | -0.99 (-1.51, -0.46) | p=0.0002 | 711 | 91% | Poor |
| 1 | Yuzhao Dai | 2020 | 7 | AST | -3.02 (-7.14,1.11) | p=0.15 | 306 | 29% | Poor |
| 2 | Kathryn J. Potter | 2025 | 4 | AST | -7.8 (-15.49,-0.12) | p=0.003 | 328 | 74% | Poor |
| 3 | Lixuan Fang | 2024 | 19 | AST | -5.22 (-9.07,-1.37) | p=0.008 | 2178 | 96% | Weak |
| 4 | Adnan Malik | 2020 | 5 | AST | -1.99 (-5.7,1.72) | p=0.29 | 180 | 43% | Poor |
| 5 | Tiantian Song | 2021 | 2 | AST | -1.31 (-3.2,0.58) | p=0.17 | 83 | 0% | Poor |
| 6 | Yuan Zhu | 2021 | 12 | AST | -2.4 (-4.55,-0.25) | p=0.03 | 551 | 49% | Poor |
| 7 | Kai Zhu | 2023 | 3 | AST | -11.44 (-17.23,-5.65) | p=0.0001 | 458 | 0% | Poor |
| 8 | Shahla Rezaei | 2021 | 12 | AST | -2.95 (-7.26,1.37) | p=0.000 | NA | 88.20% | NA |
| 9 | Alessandro Mantovani | 2020 | 6 | AST | -3.41 (-11.49,4.68) | p=0.000 | 342 | 88.20% | Poor |
| 10 | Chloe Wong | 2021 | 12 | AST | -2.92 (-8.15,2.31) | p=0.27 | 946 | 73.31% | Poor |
| 11 | Yuan-Yuan Xu | 2024 | 13 | AST | -1.09 (-2.79,0.62) | p=0.21 | 690 | 92% | Poor |
| 12 | Sanjay Bandyopadhyay | 2023 | 10 | AST | -6.89 (-9.14,-4.63) | p<0.00001 | 1334 | 91% | Recommended |
| 13 | Yan Zhao | 2023 | 10 | AST | -0.52(-1.11, 0.08) | p=0.09 | 711 | 93% | Poor |
| 14 | Wu Jianping | 2021 | 8 | AST | -3.04 (-5.93, -0.16) | p=0.04 | 283 | 96% | Poor |
| 1 | Yuzhao Dai | 2020 | 3 | GGT | -12.25 (-18.85, -5.66) | p=0.0003 | 166 | 23% | Poor |
| 2 | Lixuan Fang | 2024 | 11 | GGT | -13.68 (-16.88, -10.47) | p<0.00001 | 1726 | 61% | Recommended |
| 3 | Adnan Malik | 2020 | 5 | GGT | 5.02 (-0.86, 10.9) | p=0.09 | 180 | 45% | Poor |
| 4 | Tiantian Song | 2021 | 2 | GGT | -0.7 (-3.03, 1.63) | p=0.56 | 60 | 37% | Poor |
| 5 | Yuan Zhu | 2021 | 5 | GGT | -3.38 (-8.73, 1.96) | p=0.21 | 251 | 56% | Poor |
| 6 | Yajie Dong | 2016 | 3 | GGT | -13.82 (-20.28, -7.36) | p<0.0001 | 223 | 58% | Poor |
| 7 | Shahla Rezaei | 2021 | 5 | GGT | -11.53 (-15.21, -7.85) | p=0.071 | NA | 53.60% | NA |
| 8 | Alessandro Mantovani | 2020 | 4 | GGT | -10.17 (-14.27, -6.07) | p=0.783 | 232 | 0% | Poor |
| 9 | Chloe Wong | 2021 | 9 | GGT | -10.97 (-17.82, -4.12) | p=0.05 | 806 | 48.63% | Poor |
| 10 | Yuan-Yuan Xu | 2024 | 5 | GGT | -0.93 (-4.24, 2.38) | p=0.58 | 191 | 85% | Poor |
| 1 | Adnan Malik | 2020 | 4 | ALP | -5.16 (-11.9, 1.59) | p=0.13 | 142 | 22% | Poor |
| 2 | Tiantian Song | 2021 | 3 | ALP | -0.96 (-4.36, 2.44) | p=0.58 | 60 | NA | Poor |
| 3 | Shahla Rezaei | 2021 | 3 | ALP | -8.29 (-11.34, -5.24) | p=0.193 | NA | 39.20% | NA |
| 4 | Yuan-Yuan Xu | 2024 | 4 | ALP | -0.93 (-2.93, 1.07) | p=0.36 | 131 | 0% | Poor |
| 1 | Adnan Malik | 2020 | 4 | TC | -0.31 (-0.65,0.03) | p=0.07 | 146 | 0% | Poor |
| 2 | Shahla Rezaei | 2021 | 9 | TC | -1.17 (-5.25, 2.91) | p=0.202 | NA | 27.20% | NA |
| 3 | Deep Dutta | 2022 | 2 | TC | -5.53 (-8.45, -2.61) | p=0.0002 | 1888 | 0% | Recommended |
| 4 | Yuan-Yuan Xu | 2024 | 11 | TC | -0.1 (-0.27, 0.07) | p=0.24 | 609 | 73% | Poor |
| 5 | Sanjay Bandyopadhyay | 2023 | 9 | TC | -10.7 (-18, -3.4) | p=0.004 | 1322 | 71% | Weak |
| 6 | TANG Zhi-hui | 2017 | 4 | TC | -0.15 (-0.46, 0.12) | p=0.35 | 110 | 0% | Poor |
| 7 | Yan Zhao | 2023 | 12 | TC | -0.1(-1.56, -0.43) | p=0.0006 | 1101 | 96% | Weak |
| 8 | Wu Jianping | 2021 | 10 | TC | -0.12(-0.29, 0.05) | p=0.16 | 591 | 76% | Poor |
| 1 | Adnan Malik | 2020 | 4 | TG | -0.14 (-0.53, 0.25) | p=0.48 | 146 | 0% | Poor |
| 2 | Basile Njei | 2024 | 8 | TG | -0.12 (-0.2, -0.05) | p=0.001 | 535 | 56% | Poor |
| 3 | Shahla Rezaei | 2021 | 9 | TG | -7.07 (-17.51, 3.37) | p=0.679 | NA | 0.00% | NA |
| 4 | Deep Dutta | 2022 | 3 | TG | -21.43 (-41.63, -1.23) | p=0.04 | 1050 | 99% | Weak |
| 5 | Yuan-Yuan Xu | 2024 | 12 | TG | -0.35 (-0.61, -0.09) | p=0.009 | 612 | 87% | Poor |
| 6 | TANG Zhi-hui | 2017 | 4 | TG | -0.49 (-0.82, -0.16) | p=0.004 | 123 | 0% | Poor |
| 7 | Yan Zhao | 2023 | 13 | TG | -0.66(-0.96, -0.37) | p<0.0001 | 1137 | 97% | Recommended |
| 8 | Wu Jianping | 2021 | 8 | TG | -0.2(-0.28, 0.13) | p=0.16 | 562 | 21% | Poor |
| 1 | Adnan Malik | 2020 | 4 | LDL | -0.29 (-0.56, -0.02) | p=0.04 | 146 | 0% | Poor |
| 2 | Shahla Rezaei | 2021 | 8 | LDL | -1.67 (-10.08, 6.74) | p=0.003 | NA | 68.20% | NA |
| 3 | Deep Dutta | 2022 | 2 | LDL | -3.55 (-5.87, -1.23) | p=0.003 | 1888 | 0% | Weak |
| 4 | Yuan-Yuan Xu | 2024 | 13 | LDL | 0.03 (-0.17, 0.23) | p=0.80 | 661 | 94% | Poor |
| 5 | TANG Zhi-hui | 2017 | 4 | LDL | -0.12,(-0.45, -0.21) | p=0.48 | 110 | 8% | Poor |
| 6 | Yan Zhao | 2023 | 13 | LDL | -0.28(-0.52, -0.03) | p=0.03 | 1056 | 96% | Weak |
| 7 | Wu Jianping | 2021 | 8 | LDL | -0.18(-0.36, 0.01) | p=0.06 | 460 | 87% | Poor |
| 1 | Yuzhao Dai | 2020 | 6 | HbA1c | -0.36(-0.52, -0.19) | p<0.0001 | 342 | 0% | Poor |
| 2 | Peyman Nowrouzi-Sohrabi | 2020 | 9 | HbA1c | -0.08(-0.21, 0.04) | p=0.738 | NA | 0% | NA |
| 3 | Adnan Malik | 2020 | 5 | HbA1c | -0.62 (-0.88, -0.36) | p<0.00001 | 180 | 30% | Poor |
| 4 | Kai Zhu | 2023 | 3 | HbA1c | -0.77 (-1.18, -0.37) | p=0.01 | 440 | 75% | Poor |
| 5 | Deep Dutta | 2022 | 4 | HbA1c | -0.77 (-1.1, -0.45) | p＜0.00001 | 1117 | 100% | Recommended |
| 6 | Yuan-Yuan Xu | 2024 | 16 | HbA1c | -0.4 (-0.65, -0.15) | p=0.002 | 800 | 92% | Poor |
| 7 | Sanjay Bandyopadhyay | 2023 | 10 | HbA1c | -1.02 (-1.24, -0.8) | p＜0.00001 | 1141 | 94% | Recommended |
| 8 | TANG Zhi-hui | 2017 | 5 | HbA1c | -0.43 (-0.73, -0.13) | p=0.005 | 145 | 0% | Poor |
| 9 | Yan Zhao | 2023 | 15 | HbA1c | -0.9(-1.38, -0.42) | p=0.0002 | 1236 | 98% | Recommended |
| 1 | Yuzhao Dai | 2020 | 8 | FBG | -0.36 (-0.69, -0.03) | p=0.03 | 396 | 39% | Poor |
| 2 | Peyman Nowrouzi-Sohrabi | 2020 | 9 | FBG | -2.12 (-6.23, 1.98) | p=0.302 | NA | 15.80% | NA |
| 3 | TANG Zhi-hui | 2017 | 5 | FBG | -0.71 (-1.39, -0.03) | p=0.04 | 145 | 42% | Poor |
| 4 | Yan Zhao | 2023 | 14 | FBG | -1.35(-1.97, -0.77) | p<0.00001 | 1133 | 98.00% | Recommended |
| 1 | Peyman Nowrouzi-Sohrabi | 2020 | 6 | HOMA-IR | -0.31 (-0.59, 0.07) | p=0.000 | NA | 92.20% | NA |
| 1 | Lixuan Fang | 2024 | 6 | CRP | -0.41 (-0.78, -0.04) | p=0.03 | 386 | 70% | Poor |
| 1 | Yuzhao Dai | 2020 | 8 | Body weight | -4.58 (-8.07, -1.1) | p=0.01 | 396 | 94% | Poor |
| 2 | Lixuan Fang | 2024 | 15 | Body weight | -1.93 (-3.01, -0.85) | p=0.0005 | 863 | 93% | Poor |
| 3 | Peyman Nowrouzi-Sohrabi | 2020 | 9 | Body weight | -4.2 (-8.15, -0.25) | p=0.000 | NA | 97.60% | NA |
| 4 | Adnan Malik | 2020 | 5 | Body weight | -1.5 (-3.95, 0.l95) | p=0.23 | 180 | 79% | Poor |
| 5 | Basile Njei | 2024 | 18 | Body weight | -1.04 (-1.28, -0.81) | p<0.00001 | 1228 | 97% | Recommended |
| 6 | Kai Zhu | 2023 | 3 | Body weight | -6.53 (-11.21, -1.85) | p=0.006 | 458 | 0% | Poor |
| 1 | Kathryn J. Potter | 2025 | 3 | BMI | -1.75 (-2.35, -1.16) | p<0.00001 | 175 | 0% | Poor |
| 2 | Peyman Nowrouzi-Sohrabi | 2020 | 9 | BMI | -1.5 (-2.74, -0.39) | p=0.000 | NA | 97.80% | NA |
| 3 | Tiantian Song | 2021 | 2 | BMI | -1.01 (-1.6, -0.43) | p=0.0007 | 83 | 19% | Poor |
| 4 | Simin Fan | 2020 | 6 | BMI | -0.89 (-1.59, -0.19) | p=0.000 | NA | 20% | NA |
| 5 | Maria-Styliani Kalogirou | 2020 | 5 | BMI | -0.71 (-1.2, -0.22) | p=0.005 | 364 | 0% | Poor |
| 6 | Yuan-Yuan Xu | 2024 | 14 | BMI | -1.06 (-1.41, -0.7) | p<0.000001 | 712 | 78% | Poor |
| 7 | TANG Zhi-hui | 2017 | 5 | BMI | -1.83(-2.18, -0.58) | p=0.0008 | 145 | 0% | Poor |
| 8 | Wu Jianping | 2021 | 10 | BMI | -1.15 (-2.26, -0.04) | P=0.04 | 608 | 95% | Poor |
| 1 | Yuzhao Dai | 2020 | 8 | WC | -3.74 (-5.73, -0.74) | p=0.01 | 396 | 91% | Poor |
| 2 | Peyman Nowrouzi-Sohrabi | 2020 | 7 | WC | -4.14 (-7.09, -1.19) | p=0.000 | NA | 94.70% | NA |
| 3 | Adnan Malik | 2020 | 3 | WC | -6.19 (-14.7, 2.32) | p=0.15 | 108 | 75% | Poor |
| 4 | Wu Jianping | 2021 | 8 | WC | -3.87 (-6.62, -1.12) | P=0.006 | 486 | 92% | Poor |
| 1 | Yuan Zhu | 2021 | 5 | SAT | -28.53 (-38.07, -18.98) | p<0.000001 | 292 | 29% | Poor |
| 2 | Yuan-Yuan Xu | 2024 | 8 | SAT | -20.53 (-29.15, -11.9) | p<0.000001 | 380 | 49% | Poor |
| 1 | Yuan Zhu | 2021 | 5 | VAT | -29.05 (-42.9, -15.19) | p<0.0001 | 292 | 80% | Poor |
| 2 | Yuan-Yuan Xu | 2024 | 8 | VAT | -21.06 (-34.58, -7.55) | p=0.002 | 380 | 78% | Poor |
